# Synthesis and Immunological Evaluation of Mannosylated Desmuramyl Dipeptides Modified by Lipophilic Triazole Substituents

**DOI:** 10.3390/ijms23158628

**Published:** 2022-08-03

**Authors:** Vesna Petrović Peroković, Željka Car, Mia Bušljeta, Danijela Mihelec, Marija Paurević, Siniša Ivanković, Ranko Stojković, Rosana Ribić

**Affiliations:** 1 Department of Chemistry, Faculty of Science, University of Zagreb, Horvatovac 102a, 10000 Zagreb, Croatia; vpetrovi@chem.pmf.hr (V.P.P.); zcar@chem.pmf.hr (Ž.C.); mia.busljeta@chem.pmf.hr (M.B.); danijela.mihelec@chem.pmf.hr (D.M.); 2 Department of Chemistry, Josip Juraj Strossmayer University of Osijek, Cara Hadrijana 8/A, 31000 Osijek, Croatia; marija.paurevic@kemija.unios.hr; 3 Ruđer Bošković Institute, Bijenička cesta 54, 10000 Zagreb, Croatia; sinisa.ivankovic@irb.hr; 4 University Center Varaždin, University North, Jurja Križanića 31b, 42000 Varaždin, Croatia

**Keywords:** mannose, desmuramyl peptide, triazole, immunostimulating activity

## Abstract

Muramyl dipeptide (*N*-acetylmuramyl-L-alanyl-D-isoglutamine, MDP) is the smallest peptidoglycan fragment able to trigger an immune response by activating the NOD2 receptor. Structural modification of MDP can lead to analogues with improved immunostimulating properties. The aim of this work was to prepare mannosylated desmuramyl peptides (ManDMP) containing lipophilic triazole substituents to study their immunomodulating activities in vivo. The adjuvant activity of the prepared compounds was evaluated in the mouse model using ovalbumin as an antigen and compared to the MDP and referent adjuvant ManDMPTAd. The obtained results confirm that the α-position of D-*iso*Gln is the best position for the attachment of lipophilic substituents, especially adamantylethyl triazole. Compound **6c** exhibited the strongest adjuvant activity, comparable to the MDP and better than referent ManDMPTAd.

## 1. Introduction

**Figure 1:**
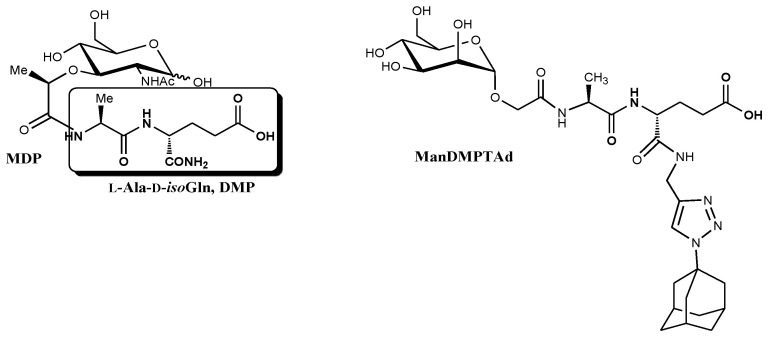
Structure of MDP, DMP and mannosylated adamantyl-triazole DMP derivative.

Peptidoglycans are polymers that constitute the cell walls of Gram-positive and Gram-negative bacteria. They act as agonists of pathogen recognition receptors (PRRs) and consequently stimulate the immune response [1,2,3]. PRRs can be generally classified as Toll-like receptors (TLRs), RIG-I-like receptors (RLRs), NOD-like receptors (NLRs) and C-type lectin receptors (CLRs) [4]. Muramyl dipeptide (MDP, *N*-acetylmuramyl-L-alanyl-D-*iso*glutamine, Figure 1) is the smallest peptidoglycan fragment (muropeptide), which enables triggering of the immune response by activating nucleotide binding oligomerization domain-containing protein 2 (NOD2). NOD2 is composed of a C-terminal leucine-rich repeat domain (LRR), a centrally positioned NOD (nucleotide-binding oligomerization domain) and two-terminally located tandem, caspase activation and recruitment domains (CARDs). Structural details of MDP binding to NOD are still lacking, but the proposed binding site is located at the LRR domain, based on the resolved crystal structure of NOD2 in the ADP-bound form [5].

Structure-activity relationship studies showed that carbohydrate moiety (muramic acid in MDP) is not essential for immunomodulation [6] and it can be replaced. MDP analogues lacking the *N*-acetylmuramyl group are called desmuramyl peptides (DMPs). Structural variations of MDP were performed to improve the pharmacological properties of the parent molecule, such as pyrogenicity and rapid elimination. It was revealed that increased lipophilicity can lead to improved immunostimulating activity [7,8,9,10,11].

Our previous research was directed toward DMPs containing lipophilic adamantane moieties attached at the N- and C-terminus of L-Ala or D-*iso*Gln and their mannosylated derivatives. Mannosylation of DMPs contributes to the stimulation of the immune response [10,12], possibly by activating mannose receptors, which are one of the PRRs [13,14]. Carbohydrate adjuvants are safe, have fewer side effects and can be used for the development of delivery systems [15]. Performed structure–activity relationship (SAR) studies of mannosylated DMPs indicated that the best position for the introduction of adamantane moiety is the C-terminus of DMP and that the glycolyl linker between mannose and peptide part is the most preferable for immunostimulating activity [12]. ManDMPTAd (Figure 1) has been identified as the most potent adjuvant in the class of mannosylated DMPs so far [16]. The immunomodulating activities of all prepared and tested compounds were evaluated in vivo in the same mouse model and based on the secondary humoral response to ovalbumin as a test antigen [12,16].

ManDMPTAd was a starting point for the development of mannosylated DMPs with improved adjuvant activity. Within this study, we have synthesized mannosylated DMPs with lipophilic substituents attached to the D-*iso*Gln/D-Glu part of dipeptide pharmacophore through a triazole structure. The immunomodulating properties of all prepared mannosylated DMPs will be assessed in a well-established mouse model and compared with the MDP and referent ManDMPTAd adjuvants.

## 2. Results

### 2.1. Chemistry

**Scheme 1:**
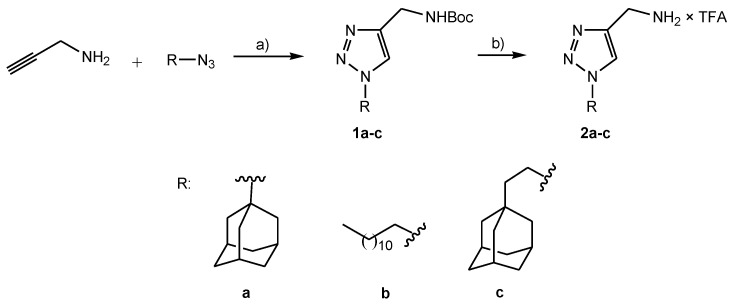
Reagents and conditions: (a): (i) Boc_2_O, TEA, 1,4-dioxane, H_2_O, 0 °C to rt, 90 min; (ii) R-N_3_, Na ascorbate (aq), CuSO_4_ × 5 H_2_O (aq), Ar, 35 °C, 24 h, yield: 97% (**1a**), 58% (**1b**), 53% (**1c**); (b) TFA, dry CH_2_Cl_2_, rt, 2–4 h; yield: 87% (**2a**), 96% (**2b**), 75% (**2c**).

**Scheme 2:**
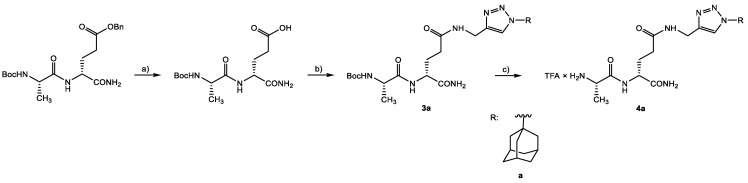
Reagents and conditions: (a) H_2_, 10% Pd/C, MeOH, 34 psi, overnight, 95%; (b) **2a**, EDC × HCl, HOBt, TEA, 1,4-dioxane/CH_2_Cl_2_, 0 °C to rt, 48 h, 71%; (c) TFA, dry CH_2_Cl_2_, rt, 2.5 h, 95%.

We prepared and characterized lipophilic triazolyl derivatives of DMP first, as well as their mannosylated analogues. The lipophilic units used were adamantan-1-yl, adamantan-1-ylethyl and dodecyl. Triazole precursors needed for coupling with DMP were prepared from corresponding lipophilic azides and propargyl amine (Scheme 1) using a one-pot reaction (Boc protection and subsequent CuAAC cycloaddition), as previously described [16]. Boc protection was then removed using standard TFA conditions and compounds Scheme 2a–c were obtained in good yields.

To incorporate **2a** into γ-COOH of the isoglutamine part of DMP, several well-known coupling methods were tested (EDC × HCl/HOBt, *n*-butyl chloroformate/*N*-methylmorpholine and HATU/DIPEA). The optimal method, as evidenced by the yield and the purity of product **3a**, was EDC × HCl/HOBt (Scheme 2). Commercially available Boc- and benzyl-protected DMP (Boc-L-Ala-D-*iso*Gln-OBn) were first deprotected using hydrogenolysis [12] and coupled with **2a**. After Boc deprotection under acidic conditions, compound **4a** was isolated.

**Scheme 3:**
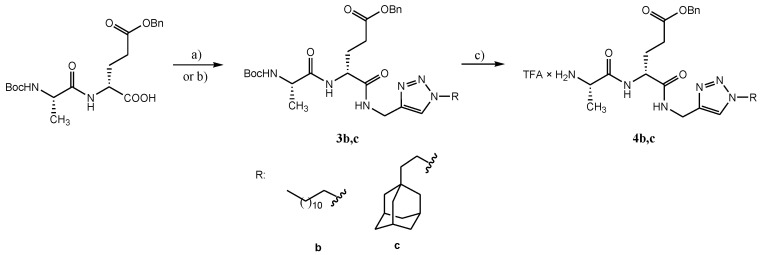
Reagents and conditions: (a) **2b**, HBTU, TEA, THF/dry CH_2_Cl_2_, Ar (g), rt, 24 h, 53% (**3b**); (b) (i) **2c**, *n*-butyl chloroformate/*N*-methylmorpholine, THF/dry CH_2_Cl_2_, Ar (g), −10 °C to rt, 3 h; (ii) NaHCO_3_ (aq), rt, 65% (**3c**); (c) TFA, dry CH_2_Cl_2_, rt, 4 h, yield: 82% (**4b**), 89% (**4c**).

Protected dipeptide (Boc-L-Ala-D-Glu-OBn), prepared according to the previously described procedure [16], was coupled with triazole derivatives **2b** and **2c** using two different coupling methods (Scheme 3), which were shown to be the most optimal for the incorporation of the corresponding lipophilic units on the α-COOH of the glutamine part of the dipeptide, mostly in terms of reaction time and yield. The *n*-Butyl chloroformate/*N*-methylmorpholine method was used for the conjugation of dipeptide with adamantylethyl-triazole derivative **2c** and HBTU/TEA coupling method was used for conjugation of dipeptide with dodecyl-triazole derivative **2b**. Boc protection was then subsequently removed and compounds **4b** and **4c** were obtained in good yields.

**Scheme 4:**
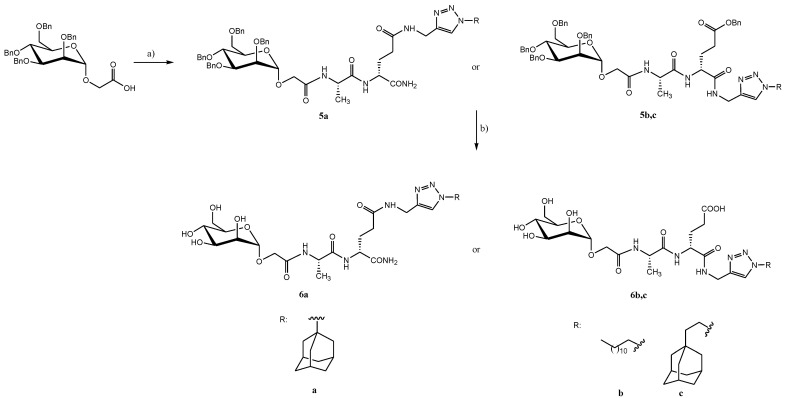
Reagents and conditions: (a) **4a–c**, EDC × HCl, HOBt, TEA, 1,4-dioxane/CH_2_Cl_2_, 0 °C to rt, 48 h, yield: 60% (**5a**), 69% (**5b**), 57% (**5c**); (b) H_2_, 10% Pd/C, 96% EtOH, 40 psi, rt, 24 h, yield: 60% (**6a**), 76% (**6b**), 67% (**6c**).

The syntheses of amide mannose conjugates **5a–c** were performed using the EDC/HOBt coupling method with TEA as a base from a previously prepared benzylated mannose precursor [16] and TFA salts **4a–c** (Scheme 4). Debenzylation of **5a–c** by catalytic hydrogenolysis gave the final products **6a–c**, unequivocally identified by ^1^H NMR and ^13^C NMR spectroscopies and mass spectrometry.

### 2.2. Immunological Evaluation

**Figure 2:**
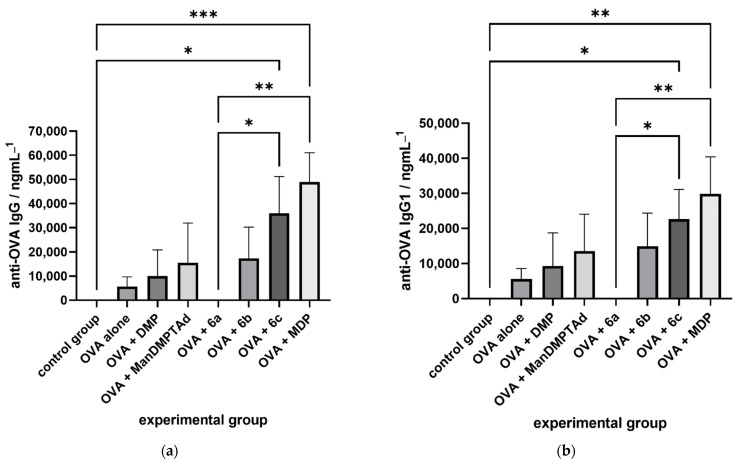
The effect of synthesized DMPs on the production of total anti-OVA IgG (**a**) and subtype of anti-OVA IgG1 (**b**) in BALB/c mice immunized with OVA as an antigen. Bar graphs represent the average values of individual mice from each group (*n* = 5). * *p* < 0.05, ** *p* < 0.01 and *** *p* < 0.001 denote statistical significance between indicated groups.

**Figure 3:**
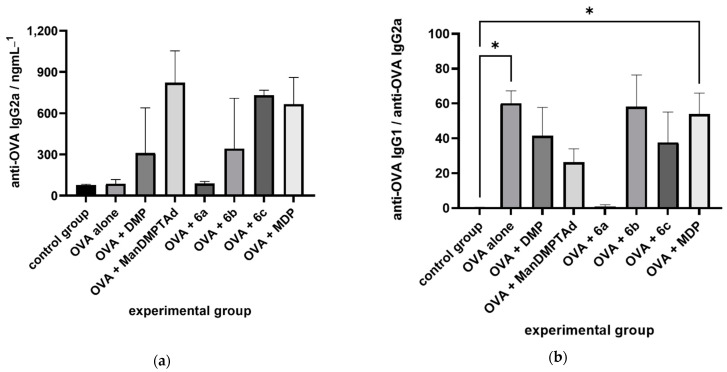
The effect of synthesized DMPs on the production of the anti-OVA IgG2a subtype (**a**) and IgG1/IgG2a ratio (**b**) in BALB/c mice immunized with OVA as an antigen. Bar graphs represent the average values of individual mice from each group (*n* = 5). * *p* < 0.05 denotes statistical significance between indicated groups.

The immunostimulating activity was estimated by the immune effect on the secondary humoral response to a well-established model antigen ovalbumin (OVA) in BALB/c mice according to previously described in vivo studies [12,17]. Evaluation of the adjuvant activity was primarily estimated based on the amount of total anti-OVA IgG antibody production (Figure 2a) after the second booster, and the immunomodulating properties were evaluated based on the amounts of produced subclasses of IgG antibodies: anti-OVA IgG1 (Figure 2b) and anti-OVA IgG2a (Figure 3a). These subclasses of IgG antibodies were measured in the mice sera because they can be used as indicators of the Th1 and Th2 of the immune response. The immune activity of the newly synthesized compounds was compared to the DMP, MDP and referent triazole compound ManDMPTAd.

Even though immunization with OVA alone in BALB/c mice model often results in high levels of IgG antibody leading to a relatively weak stimulation of total antibody production in the tested compound-injected groups [16], it can be seen that in general an enhancement in total anti-OVA IgG antibody production was observed in all groups (Figure 2a) except in the experimental group treated with compound **6a** where immunosuppression can be observed. Mice in this group were treated with compound **6a**, which is the only derivative with adamantane-triazole substituent attached to the γ-carboxyl group of the DMP pharmacophore. All other compounds, DMP, MDP, ManDMPTAd and tested **6b,c**, elicited stronger immune responses than OVA alone. In comparison to the control group, the enhancement in total anti-OVA IgG antibody production is statistically significant for MDP (*p* < 0.001) and adamantyl-triazole derivative **6c** (*p* < 0.05). The increase of total IgG antibodies is also statistically significant for MDP (*p* < 0.01) and adamantyl-triazole derivative **6c** (*p* < 0.05), in comparison to the group treated with compound **6a**. As expected, immunization with MDP led to the highest and statistically the most significant increase in the specific IgG response (*p* < 0.001). The second-best adjuvant activity was observed in the group treated with compound **6c,** which has an adamantane substituent attached to the α-carboxyl group of the DMP through triazole and ethyl linker. Stimulation of IgG antibody production with compound **6b** containing the alkyl C12 substituent is comparable to the reference mannosylated triazole ManDMPTAd.

In order to estimate the type of the generated immune response, the isotype profiles of antigen-specific anti-OVA IgG antibodies, IgG1 (Figure 2b) and IgG2a (Figure 3b), were quantitatively determined. Namely, immune adjuvants can enhance or modulate the Th1/Th2-bias of the induced immune response. Th1/Th2-polarization of the immune response can be determined by a quantification of OVA-specific IgG1 (for activation of the Th2 type) and IgG2a (for activation of the Th1 type) and calculation of the respective IgG1/IgG2a ratio.

In Figure 2b, the distribution of measured anti-OVA IgG1 antibodies over groups is presented. In all experimental groups, the trend of IgG1 antibody production was similar to that of overall anti-OVA IgG. The highest response, which was also statistically significant, was elicited by the compound MDP (*p* < 0.01) and compound **6c** (*p* < 0.05).

The production of anti-OVA IgG2a antibodies is shown in Figure 3a. No statistically significant enhancement of IgG2a antibodies was observed. The highest levels of IgG2a were exhibited in the group immunized with ManDMPTAd, followed by **6c** and MDP. As previously mentioned, the IgG1/IgG2a ratio (Figure 3b) was calculated to indirectly determine the type of immune response. From the IgG1/IgG2a ratio it is evident that all groups treated with the tested adjuvants have higher values, indicating a slight shift toward a more pronounced Th2 type of immune response. MDP significantly (*p* < 0.05) switches the immune response toward the pronounced Th2 type, due to the predominant amount of IgG1 antibodies. Compounds **6b** and **6c** switched the immune response toward a Th2-type immune reaction comparable to the MDP, even though the polarization is not statistically significant.

### 2.3. Computational Studies

To better understand the structural requirements of mannosylated DMP derivatives, a molecular docking study was performed using the SWISS-MODEL structure of NOD2 protein with incorporated seven loops that were missing in the original crystal structure (PDB ID: 5IRN)**,** described in our previous work [18]. The docking results indicate that the synthesized DMP derivatives ManDMPTAd, **6b** and **6c** bind well to the NOD2 receptor. Their dock scores (binding energy) ranged from −8.2 to −6.7 kcal/mol (Table S1). All compounds fit into the protein in a similar orientation, and there are small differences in the binding modes. In all complexes, each part of the designed molecules; L-Ala-D-isoGln, mannose and newly incorporated triazole moiety participates in hydrogen bond interactions, which suggests a good design of new potential NOD2 ligands. The compound with the lowest binding affinity is **6b**. The free carboxyl group of *iso*Gln in ligands ManDMPTAd and **6c** makes H-bonds with the side chains of Gln776 and Lys748. In both complexes, the backbone oxygen atom of L-Ala creates two H-bonds with the side chain of Arg803. In complex ManDMPTAd-NOD2, Arg857 establishes H-bonds with a triazole moiety. This type of connection is also present in the **6b**-NOD2 complex. Furthermore, the sugar component of ManDMPTAd makes the largest number of H-bonds with protein (one with Tyr801, Lys827 and Ala855 and two with Leu828), while the mannose of **6c** as a ligand realizes two H-bonds (one with Lys827 and one with Tyr801), similar to the **6b** ligand. It is important to emphasize that in all studied complexes, Phe883 establishes sugar–aromatic CH–π stacking interactions with mannose. In addition, aromatic Phe831 and Trp887 amino acids from the described hydrophobic pocket form hydrophobic interactions with the adamantane moiety (and dodecyl substituent) of the selected ligands. Additionally, in all studied complexes, the lipophilic substituents (Ad/C_12_) of ligands interact with loop 2; the Ala246 and *iso*Gln carboxyl groups also interact with Val243.

## 3. Discussion

Peptidoglycan (murein) and its smaller fragments, such as MDP, are pathogen-associated molecular patterns (PAMPs) that can be recognized by PRRs and induce an immune response [19]. The intracellular NOD2 receptor is activated by MDP binding and consequently triggers the immune response. The development of new NOD2 agonists and antagonists is an important approach for modulating the immune system. NOD2 agonists can be used as adjuvants to trigger a specific immune response in the form of either entities alone or as part of larger constructs. Several SAR studies of NOD2 agonists have been reported, including synthetic modifications at the saccharide and peptide parts of MDP [7,8,19,20,21]. Carbohydrate moiety (muramic acid) can be replaced by different substituents [6] and increase of lipophilicity can lead to improved immunostimulating activity [7,8,9,22]. Mifamurtide (liposomal muramyl tripeptide phosphatidyl ethanolamine) is a synthetically modified MDP and a clinically used therapeutic agent [23]. In mifamurtide, phospholipids facilitate the incorporation of the peptide part into liposomes due to the lipophilicity. Therefore, the introduction of lipophilic substituents has been the mainstream of SAR studies [6]. Our research is focused on the preparation of MDP mimetics, primarily modified by lipophilic adamantane due to the fact that adamantane DMPs are devoid of the undesirable side-effect [6]. Additionally, bulky adamantane can act as a membrane anchor and can be used for the preparation of liposomes and other similar drug delivery systems [24,25,26,27]. First, we prepared DMPs in which adamantyl moieties replaced the muramic acid of MDP [22] and then they were further modified by mannosylation [10,12]. We have demonstrated that the introduction of mannose enhances the adjuvant activity of parent DMPs; therefore, within this study, only mannosylated DMPs were prepared. Their effect on the secondary humoral response to ovalbumin as a test antigen in a mouse model was estimated and compared with MDP and DMP. The immunomodulating activities of all previously described compounds of the same series of DMPs were evaluated in the same in vivo model, enabling a good comparison of all results.

The design of mannosylated compounds **6b** and **6c** is based on our earlier findings that the covalent attachment of a lipophilic substituent over the triazole substructure to the C-terminus of mannosylated DMP has a beneficial effect on immunostimulatory properties [16]. Herein, we have explored the influence of three lipophilic triazole-containing substituents (adamantyl, adamantylethyl and C_12_ alkyl) on the activity of mannosylated DMP. Bulky adamantane was selected due to its beneficial properties as well as its lipophilicity and alkyl chain because clinically important MDP derivatives (murabutide, mifamurtide, B_30_-MDP) are modified by alkyl chains of moderate length [6]. The partition coefficient between *n*-octanol and water, called the log *p* value, is a classical descriptor of lipophilicity and can be predicted using computational methods. One of them is SwissADME, a web tool that we used to estimate the lipophilicity of tested compounds [28]. Their values were as follows; −2.89 for MDP, −1.19 for ManDMPTad, −1.69 for **6a**, 0.72 for **6b** and −0.53 for **6c**. This means that ManDMPTAd, as well as compounds **6a–c**, are more lipophilic than MDP. Furthermore, it can be observed that the increase of lipophilicity for compounds **6a–c** is well correlated with their improvement of adjuvant activity. **6a** with lower lipophilicity than ManDMPTAd didn’t stimulate immune response, while **6b** and **6c** with higher log *p* values have stronger immunostimulating potency. Adamantyl, adamantylethyl and C_12_ alkyl groups were connected to the L-Ala-D-*iso*Gln pharmacophore over 1,2,3-triazole moiety, which was obtained by optimized CuI-catalyzed azide/alkyne cycloaddition (CuAAC) [9,16,29,30]. Target compounds ManDMPTAd, **6a**, **6b** and **6c** were synthesized with good overall yields. ManDMPTAd was prepared according to the published procedure, and the protocol for the preparation of **6a–c** is described here for the first time. In short, adamantyl, adamantylethyl and C_12_ alkyl triazoles **2a–c** were prepared using CuAAC methodology and then conjugated with L-Ala-D-Glu/*iso*Gln peptide. Obtained adamantyl, adamantylethyl and C_12_ alkyl DMPs **4a–b** were coupled with mannose moiety via amide bond established between Ala and free carboxyl group of corresponding mannoside. After removal of the protecting groups, target compounds **6a–c** were prepared.

Immunomodulating properties of prepared mannosylated lipophilic DMPs were explored in the BALB/c mouse model in two ways: (i) the analysis of the overall production of specific anti-OVA IgG and (ii) the study of Th1 and Th2 type immune response bias. Their immunological potentials were compared to those of DMP and MDP. Results show that all mannosylated DMPs with lipophilic-triazole substituents (ManDMPTAd, **6b** and **6c**) attached to the α-COOH group of D-Glu induce higher production of anti-OVA IgG than DMP and lower than MDP (Figure 2a). Only compound **6a** with adamantane-triazole substituent on γ-COOH position suppressed the production of overall anti-OVA IgG antibodies. Furthermore, immunosuppressive activity was observed because the amount of overall IgG was lower than in the group immunized with OVA alone. In previous research, we have indicated that substitution of γ-COOH with adamantane reduces immunostimulation [12], and now we have confirmed that free γ-COOH of D-Glu/*iso*Gln is crucial for immunostimulation. On the other hand, the introduction of lipophilic-triazole moiety at α-COOH of D-Glu/*iso*Gln has a beneficial effect on immunostimulating activity. The most potent compound was **6c**, with the adamantylethyl group, followed by **6b** (C_12_ alkyl) and ManDMPTAd. Enhancement of total anti-OVA IgG antibody production by **6c** and MDP was statistically significant (*p* < 0.01 for **6c** and *p* < 0.001 for MDP). Evaluation of the **6c** compound showed the desired improvement of in vivo immunological potency relative to starting ManDMPTAd and comparable to that of MDP. The average amount of overall anti-OVA IgG antibodies in the group immunized by **6c** was only 1.36-fold lower than that for MDP. The reduction for **6b** (relative to MDP) was 2.83-fold, for ManDMPTAd, it was 3.16-fold, and for DMP, it was 4.86-fold. These results show that the lipophilic triazole structure on the α-COOH of D-*iso*Gln has a beneficial effect on immunostimulation and that adamantane substituent is a good choice for the amplification of adjuvant activity. Additionally, our research also indicated that the introduction of alkyl linker between adamantyl and triazole moieties is useful and therefore derivatives with longer alkyl linker will be examined in future research. Other research groups have also investigated MDP derivatives with substituted triazole moieties [9,19]. Modification of the C4 position of MurNAc with triazole resulted in lower affinity toward NOD2 [19], while lipophilic MDP–antigen conjugate with connected MDP and antigen part over triazole substructure showed improved immunostimulatory activity [9].

Anti-OVA IgG1 (Figure 2b) and IgG2a (Figure 3a) antibody levels and their ratio reflect the polarization of the immune reaction. For all tested compounds, the trend of IgG1 antibody production was similar to that of overall anti-OVA IgG. The highest response was elicited by the compound MDP (*p* < 0.01) and compound **6c** (*p* < 0.05). The strongest stimulation of anti-OVA IgG2a was observed in the group treated with ManDMPTAd. This result is in accordance with the literature data [16]. Based on the IgG1/IgG2a ratio (Figure 3b), it is evident that the strongest adjuvants (**6b** and **6c**) switch immune reactions toward the Th2 direction, as well as MDP (*p* < 0.05). It is well known that MDP predominantly induces IgG1 antibody production and hence stimulates the Th2-polarized immune response [17,31]. The introduction of adamantane in mannosylated DMP leads to a slightly higher production of IgG2 relative to MDP and, consequently, a reduced IgG1/IgG2a ratio, indicating that mannosylated DMP containing adamantan-triazole exhibits a more balanced Th1/Th2 immune response than MDP. Polarization of the immune reaction can also be modulated using different formulations, such as liposomes [32,33]. Jakopin et al. showed that a lipophilic DMP NOD2 agonist, after encapsulation into liposomes, induced higher levels of IgG2a antibodies [21]. Namely, entrapment of MDP into liposomes is difficult because of the lower entrapment efficiency and retention [34], while efficient encapsulation of lipophilic DMPs has been described [21,24].

Since the described glycopeptides have L-Ala-D-*iso*Gln, pharmacophore crucial for NOD2 activation and immune response triggering, we have performed docking with the most active mannosylated DMPs (ManDMPTAd, **6b**, **6c**) to investigate their NOD2 binding modes and compare them with MDP binding. The crystal structure of NOD2 revealed that Arg803, Phe831, Arg857, Trp887, Trp911, Val915 and Cys941 form the inner wall of the binding site, whereas Gly885 and Ser913 form the floor [5]. Ala substitutions proved that Phe831, Arg857, Trp887, Trp911, Gly885 and Ser913 are crucial for MDP binding [5]. Maharana et al. identified the zebrafish (Danio rerio) LRR domain binding pocket and revealed that Arg845 of zebrafish NOD2, which corresponds to Arg857 of rabbit NOD2, is found to be essential for the strong interaction of MDP, while the hydrophobic pocket composed of residues Phe819 (Phe831 in rabbit NOD2), Phe871, Trp875 (Trp887 in rabbit NOD2), Trp929 and Trp899 (Trp911 in rabbit NOD2) are responsible for correct orientation of the MDP [35]. In our previous study of interactions between MDP and structure of NOD2 protein with incorporated seven loops that were missing in the original crystal structure (PDB ID: 5IRN) [5], interactions of the dipeptide of MDP with aromatic residues (Phe831 and Trp887), which form the hydrophobic pocket, and arginines (Arg803 and Arg857) were observed [18]. Gobec et al. showed that Arg857 forms essential H-bonds with both the dipeptide of MDP and the sugar MurNAc part [7,36,37]. In addition, Lauro et al. have shown that in human NOD2, Arg877 (Arg857 in rabbit NOD2) forms critical H-bond interactions with both the carbohydrate portion of MDP (2′-*N*-acetyl group of sugar part) and the dipeptide [38]. They emphasize that mutation of this residue results in the greatest decrease in NOD2 affinity.

**Figure 4:**
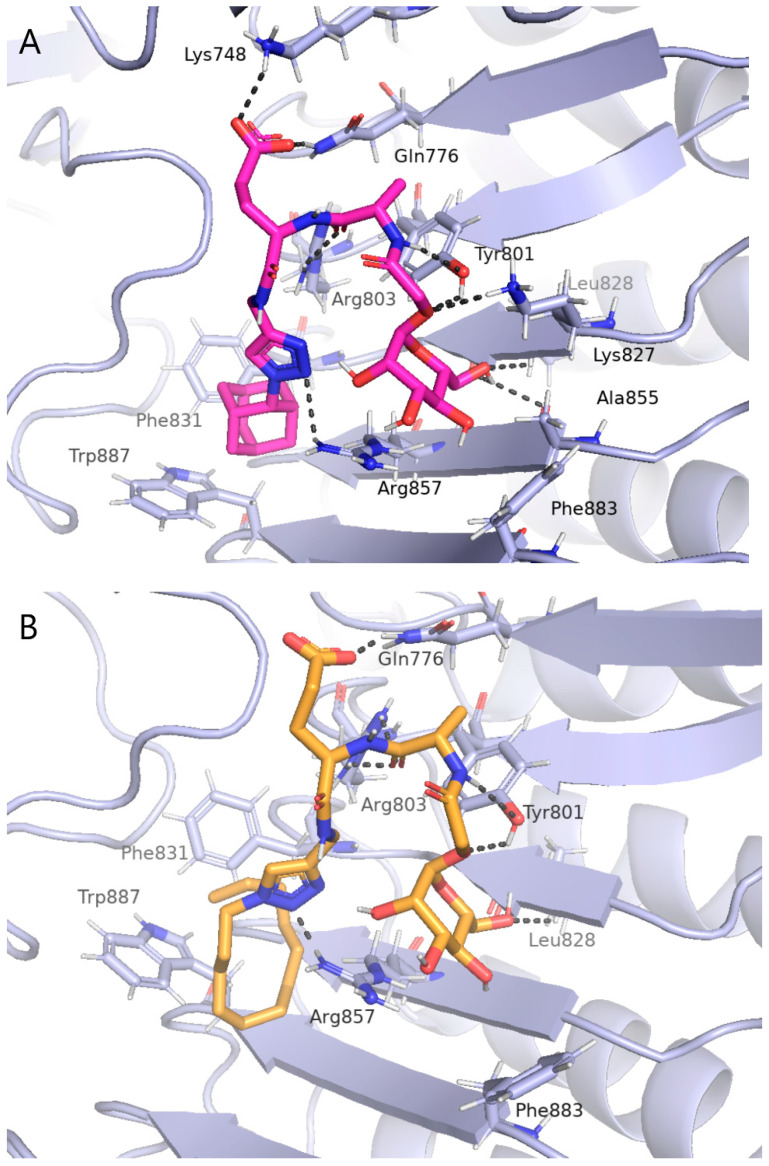

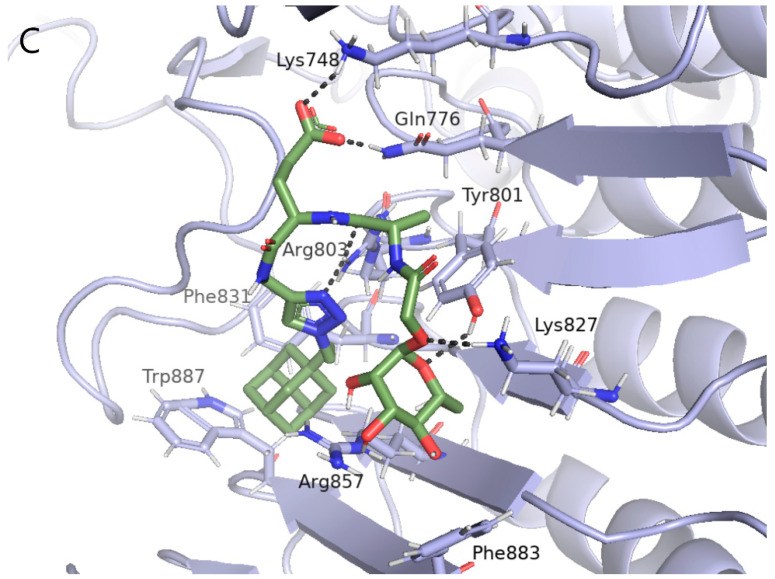
Molecular docking of ManDMPTAd (**A**), **6b** (**B**) and **6c** (**C**) in the proposed binding pocket on the concave surface of the LRR domain of NOD2. The stick model of different colors shows selected ligands, and the key amino acid residues of the NOD2 receptor around compounds have been displayed in the light blue stick model. Hydrogen bonds are shown as dark gray dashed lines.

The results of docking studies show that the binding energies of all DMPs are comparable, and they interact with the crucial residues of NOD2. They adopt different conformations, which consequently lead to their different modes of interaction with the receptor. Structural insight into the binding mode of newly synthesized DMPs highlighted the importance of every moiety: (i) mannose, (ii) the dipeptide moiety (L-Ala-D-*iso*Gln), and (iii) triazole structure modified by lipophilic groups (adamantane, alkyl chain). Every substructure contributes to overall binding. In all studied complexes (Figure 4), mannose makes H-bonds with Lys827 and Tyr801 and establishes sugar–aromatic CH–π stacking interactions. Both amino acids from DMP pharmacophore contribute to the binding; the free carboxyl group of *iso*Gln in ligands establishes two strong H-bonds with the side chains of Gln776 and Lys748, while L-Ala interacts with the side chain of Arg803. Triazole moiety is very important for NOD2 binding because it makes an H-bond with Arg857 residue, which is crucial for NOD2 activation, while lipophilic substituents form hydrophobic interactions with aromatic Phe831 and Trp887, which come from the hydrophobic pocket. Additionally, the results confirmed the importance of loop 2 in ligand recognition, as described in a previous study [18]. Figure 4 shows the difference in the binding of adamantane from **6c** and C_12_ chain from **6b**; adamantane, with its rigid structure, is deeper fitted into the hydrophobic pocket than the coiled C_12_ chain. The obtained computational results are in accordance with the results of the in vivo experiment and can be used for the design of novel DMPs with a higher binding affinity to the NOD2 receptor and improved biological activities.

Furthermore, muropeptides can act synergistically. For example, NOD2 ligands can augment the adjuvant activity of TLR ligands and modulate innate and adaptive immune responses by NLR/TLR crosstalk [3,39,40,41]. The application of chimeric agonists and multi-PRR activation represents a prospective approach in the design of vaccines [42,43]. Glycopeptide **6c** is the most potent adjuvant in the class of mannosylated DMPs and its activity is comparable with that of the MDP. There is no clear evidence that mannosylated DMPs activate only NOD2 or other PRRs, such as mannose receptors (MRs). MRs are soluble and transmembrane receptors in the CLR family [4]. Therefore, chimeric NOD2/MR agonist could also affect the immune response. Further research on this possible NOD2/MR crosstalk is required.

## 4. Materials and Methods

### 4.1. Materials

Reagents and solvents for the synthesis of compounds were obtained from Sigma-Aldrich Corp. (Darmstadt, Germany). Organic solvents were further purified and/or dried using standard methods. Thin layer chromatography (TLC) was performed on Fluka silica gel (60 F254) plates (0.25 mm). Visualization was achieved using UV light at 254 nm, 10% sulfuric acid and ninhydrine. Column chromatography was performed on Merck silica gel 60 (size 70–230 mesh ASTM). Mass spectra (ESI-MS) were recorded on an Agilent 6410 MS instrument. ^1^H and ^13^C NMR spectra of all precursors were recorded on a Bruker AV-III HD Bruker spectrometer at 400 MHz (^1^H) and 100 MHz (^13^C). All NMR experiments were performed at 298 K. Chemical shifts were referenced with respect to tetramethylsilane (TMS). HRMS spectra of the final compounds (**6a–c**) were performed on an Agilent 6550 Series Accurate-Mass Quadrupole Time-of-Flight (Q-TOF) LC/MS Agilent 1290 Infinity II with Zorbax Eclipse Plus C18 (3.0 × 50 mm, 1.8 µm) column and gradient elution (solvent A: 0.1% HCOOH in water; solvent B: 0.1% HCOOH in acetonitrile).

### 4.2. Synthesis

#### 4.2.1. Preparation of Boc Protected Lipophilic Triazole Derivatives **1a–c**

Compounds **1a–c** were prepared according to the previously described procedure [16].

##### *tert*-Butyl-[1-(adamantan-1-yl)-1*H*-1,2,3-triazol-4-yl]methyl carbamate (**1a**)

Yield 97%. Colorless oil. *R*_f_ = 0.31 (DCM/EtOAc, 3:1). ^1^H NMR (CDCl_3_) *δ* 7.60 (s, 1H, CH, triazole); 5.13 (br s, 1H, NH); 4.41 (d, 2H, *J* = 5.3 Hz, CH_2_, triazole linker); 2.27–2.23 (m, 9H: 6H, H-α, 3H, H-β); 1.83–1.74 (m, 6H, H-γ); 1.46 (s, 9H, 3 × CH_3_, Boc). ^13^C NMR (CDCl_3_) *δ* 155.9 (C, triazole); 79.6 (C, Boc); 59.6 (CH_2_, triazole linker); 43.0 (CH_2_ α); 36.2 (C, Ad); 35.9 (CH_2_ γ); 29.4 (3 × CH_3_, Boc); 28.4 (CH β). The NMR spectra are shown in Figure S1. ESI-MS: *m*/*z* [M + H]^+^ calcd for C_18_H_28_N_4_O_2_: 332.2, found: 333.1.

##### *tert*-Butyl-[1-(dodecan-1-yl)-1*H*-1,2,3-triazol-4-yl]methyl carbamate (**1b**)

Yield: 58%. Light brown solid, mp 73.0–74.5 °C. *R*_f_ = 0.25 (DCM/EtOAc, 3:1). ^1^H NMR (CDCl_3_) *δ*/ppm: 7.53 (s, 1H, CH, triazole); 5.16 (s, 1H, NH); 4.39 (s, 2H, CH_2_, triazole linker); 4.31 (t, 2H, *J* = 7.2 Hz, CH_2_, C1, dodecyl); 1.90–1.87 (m, 2H, CH_2_, C2, dodecyl); 1.43 (s, 9H, 3 × CH_3_, Boc); 1.31–1.25 (m, 18H, 9 × CH_2_, dodecyl); 0.87 (t, 3H, *J* = 7.0 Hz, CH_3_, dodecyl). ^13^C NMR (CDCl_3_) *δ*/ppm: 155.9 (C, triazole); 79.6 (C, Boc); 50.4 (C1, dodecyl); 36.1 (CH_2_, triazole linker); 31.9 (C2, dodecyl); 30.2, 29.7, 29.7, 29.6, 29.5, 29.4, 29.3, 29.0, 26.5 (C3-C10, dodecyl); 28.4 (3 × CH_3_, Boc); 22.6 (C11, dodecyl); 14.1 (C12, dodecyl). The NMR spectra are shown in Figure S2. ESI-MS: *m*/*z* [M + H]^+^ calcd for C_20_H_38_N_4_O_2_: 366.3, found: 367.3.

##### *tert*-Butyl-{1-[(2-(adamantan-1-yl)ethan-1-yl]-1*H*-1,2,3-triazol-4-yl}methyl carbamate (**1c**)

Yield 53%. Colorless oil. *R*_f_ = 0.29 (DCM/EtOAc, 3:1). ^1^H NMR (CDCl_3_) *δ*/ppm: 7.52 (s, 1H, CH, triazole); 5.13 (s, 1H, NH); 4.38 (d, 2H, *J* = 5.6 Hz, CH_2_, triazole linker); 4.35–4.32 (m, 2H, CH_2_, ethyl); 1.99 (s, 3H, H-β); 1.75–1.63 (m, 8H: 6H, H-α; 2H, CH_2_, ethyl); 1.55 (d, 6H, *J* = 2.4 Hz, H-γ); 1.44 (s, 9H, 3 × CH_3_, Boc). ^13^C NMR (CDCl_3_) *δ*/ppm: 155.9 (C, triazole); 79.6 (C, Boc); 45.8, 44.6 (2 × CH_2_, ethyl); 42.1 (CH_2_ α); 36.9 (CH_2_ γ); 36.1 (CH_2_, triazole linker); 31.97 (C, Ad); 28.4 (CH β); 28.4 (3 × CH_3_, Boc). The NMR spectra are shown in Figure S3. ESI-MS: *m*/*z* [M + H]^+^ calcd for C_20_H_32_N_4_O_2_: 360.3, found: 361.2.

#### 4.2.2. Preparation of Deprotected Lipophilic Triazole Derivatives **2a–c**

Compounds **2a–c** were prepared according to the previously described procedure [16].

##### [1-(adamantan-1-yl)-1H-1,2,3-triazol-4-yl]methylammonium trifluoroacetate (**2a**)

Yield 87%. Light brown solid, mp 98–100 °C. *R*_f_ = 0.65 (DCM/MeOH, 3:1). ^1^H NMR (CDCl_3_) *δ* 7.50 (s, 1H, CH, triazole), 3.99 (s, 2H, CH_2_, triazole linker), 2.22–2.25 (m, 9H: 6H, H-α, 3H, H-β), 1.82–1.74 (m, 6H, H-γ), 1.62 (s, 2H, NH_2_).^13^C NMR (CDCl_3_) *δ* 117.6 (CH, triazole), 59.5 (CH_2_, triazole linker), 43.0 (CH_2_ α), 35.9 (CH_2_ γ), 29.5 (CH β). The NMR spectra are shown in Figure S4. ESI-MS: *m*/*z* [M]^+^ calcd for C_13_H_21_N_4_: 233.2, found: 233.3.

##### [1-(dodecan-1-yl)-1*H*-1,2,3-triazol-4-yl] methylammonium trifluoroacetate (**2b**)

Yield 96%. Crude foam. *R*_f_ = 0.55 (CHCl_3_/MeOH, 3:1). ^1^H NMR (DMSO-*d_6_*) *δ*/ppm: 8.14 (s, 1H, CH, triazole); 4.37 (t, 2H, *J* = 7.0 Hz, CH_2_, C1, dodecyl); 4.10 (s, 2H, CH_2_, triazole linker); 1.82–1.77 (m, 2H, CH_2_, C2, dodecyl); 1.23 (s, 18H, 9 × CH_2_, dodecyl); 0.85 (t, 3H, *J* = 7.0 Hz, CH_3_). ^13^C NMR (DMSO-d6) *δ*/ppm: 124.1 (CH, triazole); 49.4 (C1, dodecyl); 33.9 (CH_2_, triazole linker); 31.2 (C2, dodecyl); 29.7, 28.9, 28.8, 28.6, 28.3, 25.7 (C3-C10, dodecyl); 22.0 (C11, dodecyl); 13.9 (C12, dodecyl). The NMR spectra are shown in Figure S5. ESI-MS: *m*/*z* [M]^+^ calcd for C_15_H_31_N_4_: 267.3, found: 267.2.

##### {1-[2-(adamantan-1-yl)ethan-1-yl]-1*H*-1,2,3-triazol-4-yl}methylammonium trifluoroacetate (**2c**)

Yield 75%. White solid, mp 147–148 °C. *R*_f_ = 0.83 (CHCl_3_/MeOH, 3:1). ^1^H NMR (DMSO-*d_6_*) *δ*/ppm: 8.23 (s, 3H, NH_3_^+^); 8.15 (s, 1H, CH, triazole); 4.43–4.38 (m, 2H, CH_2_, ethyl); 4.11 (s, 2H, CH_2_, triazole linker); 1.94 (s, 3H, H-β); 1.70–1.58 (m, 8H: 6H, H-α; 2H, CH_2_, ethyl); 1.52 (d, 6H, *J* = 2.2 Hz, H-γ). ^13^C NMR (DMSO-*d_6_*) *δ*/ppm: 124.1 (CH, triazole); 45.0, 40.0 (2 × CH_2_, ethyl); 41.3 (CH_2_ α); 36.3 (CH_2_ γ); 33.9 (CH_2_, triazole linker); 31.43 (C, Ad); 27.8 (CH β). The NMR spectra are shown in Figure S6. ESI-MS: *m*/*z* [M]^+^ calcd for C_15_H_25_N_4_: 261.2, found: 267.2.

#### 4.2.3. Preparation of Lipophilic Triazole Derivatives of Protected Desmuramyl Dipeptides **3a–c**

##### *N*-[(adamantan-1-yl)-1*H*-1,2,3-triazole-4-yl]methyl-(4*R*)-4-[(2*S*)-2-(*tert*-butyloxycarbonylamino)]propanamido}-4-carbamoylbutanoate (**3a**)

Deprotected dipeptide [12] (50 mg, 0.158 mmol) was dissolved in a mixture of dry solvents (DCM/1,4-dioxane, 1: 1; *V* = 6 mL). It was cooled to 0 °C and HOBt (21.3 mg, 0.158 mmol), as well as EDC × HCl (36.3 mg, 0.189 mmol), were added next. After 5 min, compound **2a** (1.1 equiv.) and TEA (43.9 µL, 0.315 mmol) were added and the reaction mixture was stirred at the same temperature for 1 h and then at room temperature for an additional 48 h. The reaction was monitored with TLC (CHCl_3_/MeOH, 5:1). EtOAc was added (20 mL) to the reaction mixture after reaction completion and was extracted with HCl (10 mL, *c* = 0.5 M) and then with a saturated aqueous solution of NaHCO_3_ (10 mL). The organic layer was dried over anhydrous Na_2_SO_4_. It was filtered off and the solvents were evaporated in vacuo. The crude product was purified using column chromatography with isocratic elution (CHCl_3_/MeOH, 10:1).

59.4 mg (71%). Colorless oil. *R*_f_ = 0.34 (CHCl_3_/MeOH, 10:1). ^1^H NMR (CDCl_3_) *δ*/ppm: 7.65 (s, 1H, CH, triazole); 4.59–4.41 (m, 3H: CH_2_, triazole linker, CH, *iso*Gln,); 4.17–4.11 (m, 1H, CH, Ala); 2.39–2.32 (m, 2H, CH_2_, *iso*Gln); 2.24 (s, 3H, H-β); 2.21 (s, 6H, H-γ); 2.15 (m, 1H, CH_2_, *iso*Gln); 2.09–2.00 (m, 1H, CH_2_, *iso*Gln); 1.81–1.74 (m, 6H, H-α); 1.40 (s, 9H, 3 × CH_3_, Boc); 1.34 (d, 3H, *J* = 7.1 Hz, CH_3_, Ala).^13^C NMR (CDCl_3_) *δ*/ppm: 174.3, 173.8, 173.0 (3 × C=O); 155.9 (C=O, Boc); 143.8 (C, triazole); 118.6 (CH, triazole); 80.1 (C, Boc); 59.7 (CH_2_, triazole linker); 52.4 (CH, *iso*Gln); 50.7 (CH, Ala); 42.9 (CH_2_ α); 35.9 (CH_2_ γ); 35.0 (C, Ad); 32.3 (CH_2_, *iso*Gln); 29.4 (CH β); 28.3 (3 × CH_3_, Boc); 28.1 (CH_2_, *iso*Gln); 18.1 (CH_3_, Ala). The NMR spectra are shown in Figure S7. ESI-MS: *m*/*z* [M + H]^+^ calcd for C_26_H_41_N_7_O_5_: 531.3, found: 532.1.

##### Benzyl-(4*R*)-4-{1-[(dodecan-1-yl)-1*H*-1,2,3-triazol-4-yl]methylaminocarbonyl}-4-[(2*S*)-2-(*tert*-butyloxycarbonylamino)propanamido]butanoate (**3b**)

Boc-L-Ala-D-Glu-OBn (147 mg, 0.359 mmol) was dissolved in a mixture of dry solvents (DCM: THF, 1: 1; *V* = 6 mL). After that, compound **2b** (164 mg, 0.431 mmol), previously dissolved in DCM: THF (1 mL), HBTU (259 mg, 0.68 mmol) and TEA (95 μL, 0.68 mmol) were added. The reaction mixture was stirred in an argon atmosphere at room temperature for 24 h. The reaction was monitored by TLC (CHCl_3_/MeOH, 15:1). After reaction completion, distilled water (10 mL) was added, and the mixture was extracted with DCM (2 × 15 mL). After that, the organic layer was extracted with a saturated aqueous solution of NaCl and dried over anhydrous Na_2_SO_4_. It was filtered off and the solvents were evaporated in vacuo. The crude product was purified using column chromatography with isocratic elution (CHCl_3_/MeOH, 15:1).

125 mg, 53%. Colorless oil. ^1^H NMR (CDCl_3_) *δ*/ppm: 7.50 (s, 1H, CH, triazole); 7.43 (br s, 1H, NH); 7.36–7.32 (m, 5H, 5 × CH, Bn); 7.12 (d, 1H, *J* = 7.5 Hz, NH); 5.14–5.04 (m, 3H: 2H, CH_2_, Bn; 1H, NH); 4.55–4.40 (m, 3H: 2H, CH_2_, triazole linker; 1H, CH, *iso*Gln); 4.27 (t, 2H, CH_2_, *J* = 7.3 Hz, C1, dodecyl); 4.05–3.99 (m, 1H, CH, Ala); 2.57–2.40 (m, 2H, CH_2_, *iso*Gln); 2.24–2.17 (m, 1H, CH_2_, *iso*Gln); 2.07–1.99 (m, 1H, CH_2_, *iso*Gln); 1.88–1.83 (m, 2H, CH_2_, C2, dodecyl); 1.38 (s, 9H, 3 × CH_3_, Boc); 1.32–1.25 (m, 21H: 18H, 9 × CH_2_, C3-C11, dodecyl; 3H, CH_3_, Ala); 0.88 (t, 3H, *J* = 7.0 Hz, CH_3_, dodecyl).^13^C NMR (CDCl_3_) *δ*/ppm: 173.6, 173.3, 171.2 (3 × C=O); 155.9 (C=O, Boc); 145.0 (C, triazole); 135.8 (C, Bn); 128.7, 128.5, 128.4 (CH, Bn); 122.1 (CH, triazole); 80.6 (C, Boc); 66.8 (CH_2_, Bn) 53.0 (CH, *iso*Gln); 50.9 (CH, Ala); 50.5 (CH_2_, C1, dodecyl); 35.4 (CH_2_, triazole linker); 32.0 (CH_2_, C2, dodecyl); 30.7 (CH_2_, *iso*Gln); 30.4, 29.7, 29.5, 29.4, 29.2 (CH_2_, C3-C9, dodecyl); 28.4 (CH_3_, Boc); 27.0 (CH_2_, *iso*Gln); 26.7 (CH_2_, C10, dodecyl); 22.8 (CH_2_, C11, dodecyl); 17.9 (CH_3_, Ala); 14.3 (CH_3_, dodecyl). The NMR spectra are shown in Figure S8. ESI-MS: *m*/*z* [M + H]^+^ calcd for C_35_H_56_N_6_O_6_: 656.4, found: 657.6.

##### Benzyl-(4*R*)-4-{1-[(2-(adamantan-1-yl)ethan-1-yl)-1*H*-1,2,3-triazol-4-yl]methyl-aminocabonyl}-4-[(2*S*)-2-(*tert*-butyloxycarbonylamino)propanamido]butanoate (**3c**)

Boc-L-Ala-D-Glu-OBn (250 mg, 0.613 mmol) was dissolved in dry THF (7 mL) and the mixture was cooled to −10 °C. *n*-Buthyl chloroformate (155.5 μL, 1.23 mmol), *N*-methylmorpholine (134.8 μL, 1.23 mmol) and compound **2c** were added after 10 min. The mixture was stirred in an argon atmosphere at −10 °C for 90 min and then at room temperature for an additional 3 h. The reaction was monitored by TLC (CHCl_3_/MeOH, 15: 1). After reaction completion, a saturated aqueous solution of NaHCO_3_ (10 mL) was added and the mixture was extracted with DCM (2 × 15 mL). The organic layer was dried over anhydrous Na_2_SO_4_. It was filtered off and the solvents were evaporated in vacuo. The crude product was purified using column chromatography with isocratic elution (CHCl_3_/MeOH, 15:1).

265 mg (65%). White solid, mp 71–72 °C. *R*_f_ = 0.37 (CHCl_3_/MeOH, 15:1). ^1^H NMR (CDCl_3_) *δ*/ppm: 7.51 (s, 1H, CH, triazole); 7.45 (s, 1H, NH); 7.36–7.32 (m, 5H, 5 × CH, Bn); 7.13 (d, 1H, *J* = 7.7 Hz, NH); 5.11 (d, 2H, *J* = 3.8 Hz, CH_2_, Bn); 5.07–5.05 (m, 1H, NH); 4.54–4.43 (m, 3H: 2H, CH_2_, triazole linker; 1H, CH, *iso*Gln); 4.32–4.28 (m, 2H, CH_2_, ethyl); 4.06–4.00 (m, 1H, CH, Ala); 2.56–2.42 (m, 2H, CH_2_, *iso*Gln); 2.24–2.20 (m, 1H, CH_2_, *iso*Gln); 2.05–1.98 (m, 4H: 3H, H-β; 1H, CH_2_, *iso*Gln); 1.74–1.62 (m, 8H: 6H, H-α; 2H, CH_2_, ethyl); 1.54 (d, 6H, *J* = 2.4 Hz, H-γ); 1.38 (s, 9H, 3 × CH_3_, Boc); 1.32 (d, 3H, *J* = 7.1 Hz, CH_3_, Ala). ^13^C NMR (CDCl_3_) *δ*/ppm: 173.5, 173.1, 171.1 (3 × C=O); 144.8 (C, triazole); 135.6 (C, Bn); 128.6, 128.3 (CH, Bn); 121.9 (CH, triazole); 80.5 (C, Boc); 66.7 (CH_2_, Bn); 52.9 (CH, *iso*Gln); 50.7 (CH, Ala); 45.7, 44.3 (2 × CH_2_, ethyl); 42.1 (CH_2_ α); 36.9 (CH_2_ γ); 35.3 (CH_2_, triazole linker); 30.6 (CH_2_, *iso*Gln); 28.5 (CH β); 28.3 (3 × CH_3_, Boc); 26.9 (CH_2_, *iso*Gln); 17.9 (CH_3_, Ala). The NMR spectra are shown in Figure S9. ESI-MS: *m*/*z* [M + H]^+^ calcd for C_35_H_50_N_6_O_6_: 650.4, found: 651.8.

#### 4.2.4. Preparation of Deprotected Lipophilic Triazole Derivatives Od Desmuramyl dipeptide **4a–c**

Compounds **3a–c** were deprotected according to the previously described procedure [16].

##### (1*S*)-1-{[*N*-(1*R*)-{3-(adamantan-1-yl)-1*H*-1,2,3-triazol-4-yl]methylaminocarbonyl}-1-carbamoylpropan-1-yl]aminocarbonyl}ethylammonium trifluoracetate (**4a**)

Yield 95%. Colorless oil. *R*_f_ = 0.2 (CHCl_3_/MeOH, 3:1). ^1^H NMR (CD_3_OD) *δ*/ppm: 7.95 (s, 1H, CH, triazole); 4.43 (s, 2H, CH_2_, triazole linker); 4.38 (dd, 1H, *J* = 4.8 Hz, *J* = 9.1 Hz, CH, *iso*Gln); 3.97 (q, 1H, *J* = 7.0 Hz, CH, Ala); 2.34 (t, 2H, *J* = 7.4 Hz, CH_2_, *iso*Gln); 2.25 (s, 9H: 3H, H-β, 6H, H-γ); 2.19–2.10 (m, 1H, CH_2_, *iso*Gln); 2.01–1.91 (m, 1H, CH_2_, *iso*Gln); 1.84 (t, 6H, *J* = 14.2 Hz, H-α); 1.51 (d, 3H, *J* = 7.1 Hz, CH_3_, Ala).^13^C NMR (CD_3_OD) δ/ppm: 174.5, 173.2, 169.7 (3 × C=O); 143.8 (C, triazole); 119.4 (CH, triazole); 59.8 (CH_2_, triazole linker); 52.6 (CH, *iso*Gln); 48.9 (CH, Ala); 42.5 (CH_2_ α); 35.5 (CH_2_ γ); 34.3 (C, Ad); 31.4 (CH_2_, *iso*Gln); 29.6 (CH β); 27.6 (CH_2_, *iso*Gln); 16.2 (CH_3_, Ala). The NMR spectra are shown in Figure S10. ESI-MS: *m*/*z* [M]^+^ calcd for C_21_H_34_N_7_O_3_: 432.3, found: 432.2.

##### (1*S*)-1-{[*N*-(1*R*)-{3-benzyloxycarbonyl-1-{[1-(dodecan-1-yl)-1*H*-1,2,3-triazol-4-yl]methylaminocarbonyl}propan-1-yl]aminocarbonyl}ethylammonium trifluoracetate (**4b**)

Yield 82%. Colorless oil. *R*_f_ = 0.38 (CHCl_3_/MeOH, 15:1). ^1^H NMR (CD_3_OD) *δ*/ppm: 7.83 (s, 1H, CH, triazole); 7.35–7.30 (m, 5H, 5 × CH, Bn); 5.11 (s, 2H, CH_2_, Bn); 4.43 (s, 2H, CH_2_, triazole linker); 4.27 (3H: 2H, CH_2_, C1, dodecyl, 1H, CH, *iso*Gln); 3.60 (q, 1H, *J* = 6.9 Hz, CH, Ala); 2.44 (m, 2H, CH_2_, *J* = 7.5 Hz, *iso*Gln); 2.19–2.11 (m, 1H, CH_2_, *iso*Gln); 2.01–1.93 (m, 1H, CH_2_, *iso*Gln); 1.91–1.84 (m, 2H, CH_2_, C2, dodecyl); 1.33–1.27 (m, 21H: 18H, 9 × CH_2_, C3-C11, dodecyl; 3H, CH_3_, Ala); 0.89 (t, 3H, CH_3_, *J* = 7.0 Hz, CH_3_, dodecyl). ^13^C NMR (CD_3_OD) *δ*/ppm: 176.5, 174.3, 173.8 (3 × C=O); 146.4 (C, triazole); 137.8 (C, Bn); 129.9, 129.5, (CH, Bn); 124.4 (CH, triazole); 67.7 (CH_2_, Bn) 54.3 (CH, *iso*Gln); 51.7 (CH_2_, C1, dodecyl); 51.3 (CH, Ala); 36.0 (CH_2_, triazole linker); 33.4 (CH_2_, C2, dodecyl); 31.6 (CH_2_, *iso*Gln); 31.0, 31.0, 30.9, 30.8, 30.4 (CH_2_, C3-C9, dodecyl); 28.5 (CH_2_, *iso*Gln); 27.8 (CH_2_, C10_,_ dodecyl); 24.0 (CH_2_, C11) C_12_); 20.4 (CH_3_, Ala); 14.7 (CH_3_, dodecyl). The NMR spectra are shown in Figure S11. ESI-MS: *m*/*z* [M]^+^ calcd for C_21_H_34_N_7_O_3_: 432.3, found: 432.2.

##### (1*S*)-1-{[*N*-(1*R*)-{3-benzyloxycarbonyl-1-{[1-(2-(adamantan-1-yl)ethan-1-yl]-1*H*-1,2,3-triazol-4-yl]methylaminocarbonyl}propan-1yl]aminocarbonyl}ethylammonium trifluoracetate (**4c**)

Yield 89%. Light yellow solid, mp 64–66 °C. *R*_f_ = 0.38 (CHCl_3_/MeOH, 4:1). ^1^H NMR (CD_3_OD) *δ*/ppm: 7.86 (s, 1H, CH, triazole); 7.34–7.29 (m, 5H, 5 × CH, Bn); 5.11 (s, 2H, CH_2_, Bn); 4.43–4.36 (m, 5H: 2H, CH_2_, triazole linker; 2H, CH_2_, ethyl; 1H, CH, *iso*Gln); 3.98–3.93 (m, 1H, CH, Ala); 2.44 (t, 2H, CH_2_, *J* = 7.5 Hz, *iso*Gln); 2.19–2.10 (m, 1H, CH_2_, *iso*Gln); 2.01–1.96 (m, 4H: 3H, H-β; 1H, CH_2_, *iso*Gln); 1.78–1.64 (m, 8H: 6H, H-α; 2H, CH_2_, ethyl); 1.58 (s, 6H, H-γ); 1.48 (d, 3H, *J* = 7.0 Hz, CH_3_, Ala). ^13^C NMR (CD_3_OD) *δ*/ppm: 172.5, 171.8, 169.8 (3 × C=O); 136.1 (C, Bn); 128.2; 127.8 (CH, Bn); 66.1 (CH_2_, Bn); 52.7 (CH, *iso*Gln); 48.9 (CH, Ala); 45.5, 44.1 (2 × CH_2_, ethyl); 41.7 (CH_2_ α); 36.6 (CH_2_ γ); 34.3 (CH_2_, triazole linker); 31.6 (C, Ad); 29.8 (CH_2_, *iso*Gln); 28.6 (CH β); 26.8 (CH_2_, *iso*Gln); 16.2 (CH_3_, Ala). The NMR spectra are shown in Figure S12. ESI-MS: *m*/*z* [M]^+^ calcd for C_30_H_43_N_6_O_4_: 551.3, found: 551.7.

#### 4.2.5. Preparation of Protected Mannose Glycoconjugates of Lipophilic Triazole Derivatives of Desmuramyl Dipeptides **5a–c**

Compounds **5a–c** were prepared according to the previously described procedure [16].

##### *N*-[(adamantan-1-yl)-1*H*-1,2,3-triazole-4-yl]methyl-(4*R*)-4-{(2*S*)-2-[(2,3,4,6-tetra-*O*-benzyl-α-D-mannopyranosyloxy)ethanamido]propanamido}-4-carbamoylbutanoate (**5a**)

Yield 60%. Yellow oil. *R*_f_ = 0.22 (CHCl_3_/MeOH, 12:1). ^1^H NMR (CDCl_3_) *δ*/ppm: 7.57 (s, 1H, triazole); 7.39–7.16 (m, 20H, 20 × CH, Bn); 4.96 (d, 1H, *J*_1,2_ = 1.8 Hz, H-1); 4.86–4.62 (m, 6H: 4H, 2 × CH_2_, Bn; CH_2_, triazole linker); 4.56–4.37 (m, 6H: 4H, 2 × CH_2_, Bn; 1H, CH, Ala, 1H, CH, *iso*Gln); 4.17 (d, 1H, *J* = 15.5 Hz, CH, acetyl linker); 4.05–3.99 (m, 2H, H-2, CH, acetyl linker); 3.92 (dd, 1H, *J*_3,2_ = 8.8 Hz, *J*_3,4_ = 3.0 Hz, H-3); 3.84 (t, 1H, *J*_4,3_ = 2.5 Hz, H-4); 3.77–3.67 (m, 3H, H-6a, H-6b, H-5); 2.43–2.29 (m, 2H, CH_2_, *iso*Gln); 2.24 (br s, 3H, H-β); 2.02 (br s, 6H, H-α); 2.13–2.03 (m, 2H, CH_2_, *iso*Gln); 1.81–1.74 (m, 6H, H-γ); 1.37 (d, 3H, *J* = 7.2 Hz, CH_3_, Ala). ^13^C NMR (CDCl_3_) *δ*/ppm: 173.7, 173.1, 172.5, 169.3 (4 × C=O); 143.4 (C, triazole); 138.4, 138.3, 138.2, 138.1 (4 × C, Bn); 128.4–127.6 (CH, Bn); 118.5 (CH, triazole); 98.4 (C1); 79.4, 74.8, 74.7, 72.6 (C2-C5); 74.9, 73.4, 72.8, 72.5 (4 × CH_2_, Bn); 68.9 (C6), 66.3 (CH_2_, acetyl linker); 59.8 (CH_2_, triazole linker); 52.7 (CH, *iso*Gln); 49.1 (CH, Ala); 42.9 (CH_2_ α); 35.8 (CH_2_ γ); 35.0 (C, Ad); 32.3 (CH_2_, *iso*Gln); 29.4 (CH β); 27.6 (CH_2_, *iso*Gln); 18.1 (CH_3_, Ala). The NMR spectra are shown in Figure S13. ESI-MS: *m*/*z* [M + H]^+^ calcd for C_57_H_69_N_7_O_10_: 1011.5, found: 1012.6.

##### Benzyl-(4*R*)-4-{[1-(dodecane-1-yl)-1*H*-1,2,3-triazole-4-yl]methylaminocarbonyl}-4-(2*S*)-2-[2,3,4,6-tetra-*O*-benzyl-α-D-mannopyranosyloxy)ethanamido]propanamido}butanoate (**5b**)

Yield 69%. Yellow oil. *R*_f_ = 0.70 (9% MeOH in DCM). ^1^H NMR (CD_3_OD) *δ*/ppm: 7.72 (s, 1H, CH, triazole); 7.40–7.16 (m, 25H, 25 × CH, Bn); 5.09 (s, 2H, CH_2_, Bn ester); 4.99 (d, 1H, *J*_1,2_ = 1.8 Hz, H-1); 4.70 (s, 2H, CH_2_, triazole linker); 4.82–4.32 (m, 10H: 8H, 4 × CH_2_, Bn; 1H, CH, Ala; 1H, CH, *iso*Gln); 4.24 (t, 2H, *J* = 7.1 Hz, CH_2_, C1, dodecyl); 4.15–3.92 (m, 5H: 2H, CH_2_, acetyl linker; 3H, H-2, H-3, H-4); 3.77–3.74 (m, 1H, H-5); 3.72–3.64 (m, 2H: H-6a; H-6b); 2.44 (t, 2H, *J* = 7.3 Hz, CH_2_, *iso*Gln); 2.22–2.16 (m, 1H, CH_2_, *iso*Gln); 1.98–1.91 (m, 1H, CH_2_, *iso*Gln); 1.81–1.74 (m, 2H, CH_2_, C2, dodecyl); 1.35 (d, 3H, *J* = 7.1 Hz, CH_3_, Ala); 1.28–1.24 (m, 18H, 9 × CH_2_, C3-C11, dodecyl); 0.88 (t, 3H, *J* = 6.9 Hz, CH_3_, dodecyl). ^13^C NMR (CD_3_OD) *δ*/ppm: 178.7, 177.9, 177.1, 175.3 (4 × C=O); 149.8 (C, triazole); 143.5, 143.3, 143.2 (C, Bn); 133.2–132.3 (CH, Bn); 127.7 (CH, triazole); 103.2 (CH, C1); 84.6, 79.6, 79.4, 77.3, (CH, C2-C5); 79.5, 78.0, 77.4, 76.6 (4 × CH_2_, Bn); 73.9 (CH_2_, C6); 71.1 (CH_2_, Bn-ester); 70.8 (CH_2_, acetyl linker); 57.8 (CH, *iso*Gln); 54.9 (CH_2_, C1, dodecyl); 54.0 (CH, Ala); 39.5 (CH_2_, triazole linker); 36.7 (CH_2_, C2, dodecyl); 35.0, 34.9, 34.4, 34.3, 34.2, 34.1, 33.7, 31.3, 31.1 (CH_2_, C3-C10, dodecyl; 2 × CH_2_, *iso*Gln); 27.3 (CH_2_, C11, dodecyl); 21.5 (CH_3_, Ala); 18.1 (CH_3_, dodecyl). The NMR spectra are shown in Figure S14. ESI-MS: *m*/*z* [M + H]^+^ calcd for C_66_H_84_N_6_O_11_: 1136.6, found: 1137.5.

##### Benzyl-(4*R*)-4-{*N*-{1-[(2-(adamantan-1-yl)ethan-1-yl]-1*H*-1,2,3-triazole-4-il}methylcarbamoyl}-4-{(2*S*)-2-[(2,3,4,6-tetra-*O*-benzyl-α-D-mannopyranosyloxy)ethanamido]propanamido}butanoate (**5c**)

Yield 57%. Yellow oil. *R*_f_ = 0.49 (6% MeOH in DCM). ^1^H NMR (CD_3_OD) *δ*/ppm: 7.74 (s, 1H, triazole); 7.39–7.16 (m, 25H, 25 × CH, Bn); 5.09 (s, 2H, CH_2_, Bn ester); 4.99 (d, 1H, *J*_1,2_ = 1.3 Hz, H-1); 4.67 (s, 2H, CH_2_, triazole linker); 4.82–4.32 (m, 10H: 8H, 4 × CH_2_, Bn; 1H, CH, Ala, 1H, CH, *iso*Gln); 4.29–4.25 (m, 2H, CH_2_, ethyl); 4.15–3.90 (m, 5H: 2H, CH_2_, acetyl linker; 3H, H-2, H-3, H-4); 3.78–3.75 (m, 1H, H-5); 3.72–3.64 (m, 2H, H-6a, H-6b); 2.44 (t, 2H, *J* = 7.3 Hz, CH_2_, *iso*Gln); 2.23–2.15 (m, 1H, CH_2_, *iso*Gln); 1.98–1.91 (m, 1H, CH_2_, *iso*Gln); 1.90 (br s, 3H, H-β); 1.73–1.62 (m, 6H, H-α), 1.59–1.54 (m, CH_2_, ethyl); 1.50 (br s, 6H, H-γ); 1.35 (d, 3H, *J* = 7.1 Hz, CH_3_, Ala). ^13^C NMR (CD_3_OD) *δ*/ppm: 178.6, 177.9, 177.1, 175.3 (4 × C=O); 149.8 (C, triazole), 143.5, 143.3, 143.2 (C, Bn); 133.0–132.3 (CH, Bn); 127.6 (CH, triazole); 103.2 (C1); 84.6, 79.6, 79.4, 77.3 (C2-C5); 79.5, 78.0, 77.4, 76.6 (4 × CH_2_, Bn); 73.9 (C6), 71.1 (CH_2_, Bn-ester); 70.7 (CH_2_, acetyl linker); 57.8 (CH, *iso*Gln); 54.0 (CH, Ala); 50.4, 49.1 (2 × CH_2_, ethyl); 46.7 (CH_2_ α); 41.6 (CH_2_ γ); 39.5 (CH_2_, triazole linker); 35.0 (CH_2_, *iso*Gln); 33.6 (CH β); 31.3 (CH_2_, *iso*Gln); 21.5 (CH_3_, Ala). The NMR spectra are shown in Figure S15. ESI-MS: *m*/*z* [M + H]^+^ calcd for C_66_H_78_N_6_O_11_: 1130.6, found: 1131.4.

#### 4.2.6. Preparation of Deprotected Mannose Glycoconjugates of Lipophilic Triazole Derivatives of Desmuramyl Dipeptides **6a–c**

Compounds **5a–c** were deprotected according to the previously described procedure [16].

##### *N*-[(adamantan-1-yl)-1*H*-1,2,3-triazole-4-yl]methyl-(4*R*)-4-{(2*S*)-2-[(α-D-mannopyranosyloxy)ethanamido]propanamido}-4-carbamoylbutanoate (**6a**)

Yield 50%. Colorless oil. *R_f_* = 0.33 (CHCl_3_/MeOH, 2:1). ^1^H NMR (CD_3_OD) *δ*/ppm: 7.99 (s, 1H, triazole); 4.86 (d, 1H, *J*_1,2_ = 1.8 Hz, H-1); 4.47 (d, 2H, *J* = 3.9 Hz, CH_2_, triazole linker); 4.44–4.39 (q, 1H, *J* = 7.1 Hz, CH, Ala); 4.34 (dd, 1H, *J* = 9.5 Hz, *J* = 4.7 Hz, CH, *iso*Gln); 4.24 (d, 1H, *J*_gem_ = 15.2 Hz, CH_2_, acetyl linker), 4.12 (d, 1H, *J*_gem_ = 15.2 Hz, CH_2_, acetyl linker); 3.97 (dd, 1H, *J*_2,3_ = 3.5 Hz, *J*_1,2_ = 1.8 Hz, H-2); 3.86 (1H, dd, *J*_6a,6b_ = 11.6 Hz, *J*_5,6a_ = 2.2 Hz, H-6b); 3.79 (dd, 1H, *J*_3,4_ = 9.1 Hz, *J*_2,3_ = 3.5 Hz, H-3); 3.70 (dd, 1H, *J*_6a,6b_ = 11.6 Hz, *J*_5,6b_ = 5.7 Hz, H-6a); 3.63 (app t, 1H, *J* = 9.4 Hz, H-4); 3.59–3.54 (m, 1H, H-5); 2.35 (t, 2H, *J* = 7.2 Hz, CH_2_, *iso*Gln); 2.20–2.17 (m, 1H, CH_2_, *iso*Gln); 2.27 (br s, 9H, H-α, H-β); 2.01–1.90 (m, 1H, CH_2_, *iso*Gln); 1.85 (br s, 6H, H-γ); 1.42 (d, 3H, *J* = 7.2 Hz, CH_3_, Ala). ^13^C NMR (CD_3_OD) *δ*/ppm: 174.9, 173.5, 173.4, 170.5 (4 × C=O); 144.0 (C, triazole); 119.4 (CH, triazole); 100.3 (C1); 74.0, 71.0, 70.3, 67.2 (C2-C5); 65.5 (C6), 61.4 (CH_2_, acetyl linker); 59.7 (CH_2_, triazole linker); 56.9 (CH, *iso*Gln), 52.6 (CH, Ala); 42.5 (CH_2_ α); 35.5 (CH_2_ γ); 34.4 (C, Ad); 31.5 (CH_2_, *iso*Gln); 29.6 (CH β); 27.1 (CH_2_, *iso*Gln); 16.6 (CH_3_, Ala). The NMR spectra are shown in Figure S16. ESI-MS: *m*/*z* [M + H]^+^ calcd for C_29_H_45_N_7_O_10_: 651.3, found: 652.3. HRMS: calcd for C_29_H_46_N_7_O_10_ [M + H]^+^: 652.3308, found: 652.3306.

##### (4*R*)-4-{*N*-[1-(dodecan-1-yl)-1*H*-1,2,3-triazol-4-yl]methylcarbamoyl}-4-{(2*S*)-2-[(α-D-mannopyranosyloxy)ethanamido]propanamido}butanoic acid (**6b**)

Yield 76%. Colorless oil. *R*_f_ = 0.53 (CH_3_CN/H_2_O, 5:1). ^1^H NMR (CD_3_OD) *δ*/ppm: 7.84 (s, 1H, CH, triazole); 4.83 (d, 1H, *J*_1,2_ = 1.5 Hz, H-1); 4.45 (d, 2H, *J* = 2.6 Hz, CH_2_, triazole linker); 4.42–4.34 (m, 4H: 2H, CH_2_, C1, dodecyl; 1H, CH, Ala; 1H, CH, *iso*Gln); 4.19 (d, 1H, *J*_gem_ = 15.2 Hz, CH_2_, acetyl linker, 4.06 (d, 1H, *J*_gem_ = 15.2 Hz, CH_2_, acetyl linker); 3.95 (dd, 1H, *J*_2,3_ = 3.3 Hz, *J*_1_,_2_ = 1.7 Hz, H-2); 3.84 (1H, dd, *J*_6a,6b_ = 11.8 Hz, *J*_5,6a_ = 2.0 Hz, H-6b); 3.79 (dd, 1H, *J*_3,4_ = 9.0 Hz, *J*_2,3_ = 3.4 Hz, H-3); 3.69 (dd, 1H, *J*_6a,6b_ = 11.8 Hz, *J*_5,6b_ = 5.8 Hz, H-6a); 3.61 (app t, 1H, *J* = 9.4 Hz, H-4); 3.59–3.55 (m, 1H, H-5); 2.35 (t, 2H, *J* = 6.9 Hz, CH_2_, *iso*Gln); 2.19–2.12 (m, 1H, CH_2_, *iso*Gln); 1.98–1.86 (m, 3H: 2H, CH_2_, C2, dodecyl; 1H, CH_2_, *iso*Gln); 1.40 (d, 3H, *J* = 7.1 Hz, CH_3_, Ala); 1.32–1.28 (m, 18H, CH_2_, C3-C11, dodecyl); 0.89 (t, 3H, *J* = 6.9 Hz, CH_3_, dodecyl). ^13^C NMR (CD_3_OD) *δ*/ppm: 173.6, 172.4, 170.4 (4 × C=O); 144.3 (C, triazole); 122.8 (CH, triazole); 100.4 (C1); 73.9, 71.0, 70.3, 67.2 (C2-C5); 65.5 (C6); 61.6 (CH_2_, acetyl linker); 53.2 (CH, *iso*Gln); 50.0 (CH_2_, C1, dodecyl); 49.0 (CH, Ala); 34.4 (CH_2_, triazole linker); 31.7 (CH_2_, C2, dodecyl); 30.7–26.1 (CH_2_, C3-C10, dodecyl; 2 × CH_2_, *iso*Gln,); 22.3 (CH_2_, C11, dodecyl); 16.6 (CH_3_, Ala), 13.0 (CH_3_, dodecyl). The NMR spectra are shown in Figure S17. ESI-MS: *m*/*z* [M + H]^+^ calcd for C_31_H_54_N_6_O_11_: 686.4, found: 687.3. HRMS: calcd for C_31_H_55_N_6_O_11_[M + H]^+^: 687.3931, found: 687.3929.

##### (4*R*)-4-{*N*-{1-[(2-(adamantan-1-yl)ethan-1-yl]-1*H*-1,2,3-triazol-4-yl}methylcarbamoyl}-4-{(2*S*)-2-[(α-D-mannopyranosyloxy)ethanamido]propanamido}butanoic acid (**6c**)

Yield 67%. Colorless oil. *R*_f_ = 0.30 (CH_3_CN/H_2_O, 5:1). ^1^H NMR (CD_3_OD) *δ*/ppm: 7.86 (s, 1H, triazole); 4.83 (d, 1H, *J*_1,2_ = 1.7 Hz, H-1); 4.45 (s, 2H, CH_2_, triazole linker); 4.42–4.38 (m, 3H: 1H, CH, Ala; 2H, CH_2_, ethyl); 4.37–4.33 (m, 1H, CH, *iso*Gln); 4.19 (d, 1H, *J*_gem_ = 15.2 Hz, CH_2_, acetyl linker), 4.07 (d, 1H, *J*_gem_ = 15.2 Hz, CH_2_, acetyl linker); 3.95 (dd, 1H, *J*_2,3_ = 3.4 Hz, *J*_1_,_2_ = 1.7 Hz, H-2); 3.84 (1H, dd, *J*_6a,6b_ = 11.8 Hz, *J*_5,6a_ = 2.1 Hz, H-6b); 3.79 (dd, 1H, *J*_3,4_ = 9.0 Hz, *J*_2,3_ = 3.4 Hz, H-3); 3.70 (dd, 1H, *J*_6a,6b_ = 11.8 Hz, *J*_5,6b_ = 5.7 Hz, H-6a); 3.63 (app t, 1H, *J* = 9.4 Hz, H-4); 3.60–3.55 (m, 1H, H-5); 2.36 (t, 2H, *J* = 7.1 Hz, CH_2_, *iso*Gln); 2.21–2.12 (m, 1H, CH_2_, *iso*Gln); 1.97–1.88 (br s, 4H: 3H, H-β; 1H, CH_2_, *iso*Gln); 1.78–1.65 (m, 8H: 6H, H-α; CH_2_, ethyl); 1.59 (br s, 6H, H-γ); 1.40 (d, 3H, *J* = 7.1 Hz, CH_3_, Ala).^13^C NMR (CD_3_OD) *δ*/ppm: 173.7, 172.3, 170.5 (4 × C=O); 122.8 (CH, triazole); 100.4 (C1); 73.9, 71.0, 70.3, 67.2 (C2-C5); 65.5 (C6), 61.4 (CH_2_, acetyl linker); 53.1 (CH, *iso*Gln), 49.1 (CH, Ala); 45.5, 44.1 (2 × CH_2_, ethyl); 41.7 (CH_2_ α); 36.6 (CH_2_ γ); 34.4 (CH_2_, triazole linker); 29.3 (CH_2_, *iso*Gln); 28.6 (CH β); 26.6 (CH_2_, *iso*Gln); 16.6 (CH_3_, Ala). The NMR spectra are shown in Figure S18. ESI-MS: *m*/*z* [M + H]^+^ calcd for C_31_H_48_N_6_O_11_: 680.3, found 681.2. HRMS: *m*/*z* [M + H]^+^ calcd for C_31_H_49_N_6_O_11_[M + H]^+^: 680.3361, found 681.3458.

### 4.3. Computational Studies

Three-dimensional models of the compounds ManDMPTAd, **6a**, **6b** and **6c** were built in GaussView 6 [44]. Their geometries were optimized using the semiempirical PM6 method in Gaussian 16 [44]. The binding site on the concave surface of the NOD2 LRR domain that contains important residue Arg857 was defined using GHECOM, a server for finding pockets on protein surfaces using mathematical morphology. Molecular docking calculations were performed using AutoDock Vina 1.1.2 [15]. The default parameters were used, the X, Y and Z coordinates of the pocket mass center were 58.866, 60.036, and 106.556 Å, and box dimensions were set to 25, 25, 25 Å. The docking results obtained were analyzed in Chimera [45] and PyMOL [46]. The highest ranked binding pose was used for graphical representation in PyMOL and values of the scoring functions are given in Table S1.

### 4.4. Experiments In Vivo

Healthy, nulliparous and non-pregnant BALB/c female mice, 8–12 weeks of age at the initiation of the experiment were used. Mice were obtained from the Ruđer Bošković Institute’s breeding colony. During the experimental period, groups of five animals were kept per cage. The bottom of the cage was covered with sawdust (Scobis Uno^®^, Muchedola srl, Settimo Milanese MI, Italy). Standard food for laboratory mice (4RF 21 GLP^®^ Mucedola srl, Settimo Milanese MI, Italy) was used. Access to food and water was ad libitum. Animals were kept in conventional circumstances: light/dark rhythms 12/12 h, temperature 22 °C, and humidity 55%. All experiments were performed according to the ILAR Guide for the Care and Use of Laboratory Animals, EU Directive 2010/63/EU and Croatian animal protection law (NN 102/17). Experimental groups of five mice were immunized and boosted two times subcutaneously (s.c.) into the tail base at 21-day intervals. Mice were anesthetized by Isoflurane prior to blood collection on the 7th day after the second booster. Sera were collected, decomplemented at 56 °C for 30 min and stored at −20 °C until tested. The dose of OVA (antigen) was 10 µg per mouse. The dose of PGM and the tested compounds was 200 µg per mouse. OVA and tested substances were dissolved in water and the injection volume in all experimental groups was 0.1 mL per mouse.

### 4.5. Enzyme Immunoassays

Enzyme immunoassays (ELISA) were performed on flat-bottomed high binding microtiter plates (Costar, Phoenix, Arizona, USA) using mouse anti-OVA IgG, IgG1 and IgG2a antibody assay kits (Chondrex, Woodinville, WA, USA), according to the manufacturer’s instructions. The ratios of anti-OVA IgG1 and anti-OVA IgG2a (IgG1/IgG2a) was calculated as an indication of the Th1/Th2-bias of the induced immune response.

### 4.6. Statistics

Statistical analyses were performed using GraphPad Prism Software. The significant difference between the experimental groups was evaluated by Kruskal–Wallis ANOVA, followed by Dunn’s multiple comparison test. Probability values less than 0.05 (*p* < 0.05) were considered significant.

## 5. Conclusions

The efficient synthesis of mannosylated DMPs modified by lipophilic triazole substituents was performed. The immunological properties of prepared mannosylated DMPs were evaluated in a mouse model. Compounds **6b** and **6c** showed improved immunological potency in vivo relative to starting ManDMPTAd. Compound **6c** exhibited adjuvant activity comparable to MDP and with a more balanced Th1/Th2 immune response. The obtained results confirm that the α-position of D-*iso*Gln is the best position for the attachment of lipophilic substituents, especially adamantylethyl triazole, in the class of mannosylated DMPs. Glycopeptide **6c** is the most active water-soluble adjuvant in the class of adjuvants. Therefore, it can be considered a new lead compound and a prospective candidate for future research, especially as a candidate capable of simultaneously triggering various types of receptors of the immune system.

## Data Availability

The datasets supporting the conclusions of this article are included within the article.

## Supplementary Materials

The following supporting information can be downloaded at: https://www.mdpi.com/article/10.3390/ijms23158628/s1.
