# Supplementary material for: Synthesis and Immunological Evaluation of Mannosylated Desmuramyl Dipeptides Modified by Lipophilic Triazole Substituents
Source: Int J Mol Sci. 2022 Aug 3;23(15):8628. doi: 10.3390/ijms23158628 (PMC9368957; doi:10.3390/ijms23158628)
Supplement: Supplementary file 1 [file ijms-23-08628-s001.zip › ijms-1837963-supplementary.pdf]

Supporting Information

Synthesis and Immunological Evaluation of Mannosylated Desmuramyl Dipeptides Modified by Lipophilic Triazole Substituents

Vesna Petrović Peroković [1,†], Željka Car [1,†], Mia Bušljeta [1], Danijela Mihelec [1], Marija Paurević [2], Siniša Ivanković [3], Ranko Stojković [3,*] and Rosana Ribić [4,*]

1 Department of Chemistry, Faculty of Science, University of Zagreb, Horvatovac 102a, 10000 Zagreb, Croatia; vpetrovi@chem.pmf.hr (V.P.P.); zcar@chem.pmf.hr (Ž.C.); mia.busljeta@chem.pmf.hr (M.B.); danijela.mihelec@chem.pmf.hr (D.M.)

2 Department of Chemistry, Josip Juraj Strossmayer University of Osijek, Cara Hadrijana 8/A, 31000 Osijek, Croatia; marija.paurevic@kemija.unios.hr

3 Rud¯er Boškovic´ Institute, Bijenicˇka cesta 54, 10000 Zagreb, Croatia; sinisa.ivankovic@irb.hr

4 University Center Varaždin, University North, Jurja Križani´ca 31b, 42000 Varaždin, Croatia

* Correspondence: stojkov@irb.hr (R.S.); rosana.ribic@unin.hr (R.R.);
Tel.: +385-915-141-256 (R.S.); +385-4249-3306 (R.R.)

† These authors contributed equally to this work.

1. NMR spectra (^{1}H, DEPTQ)

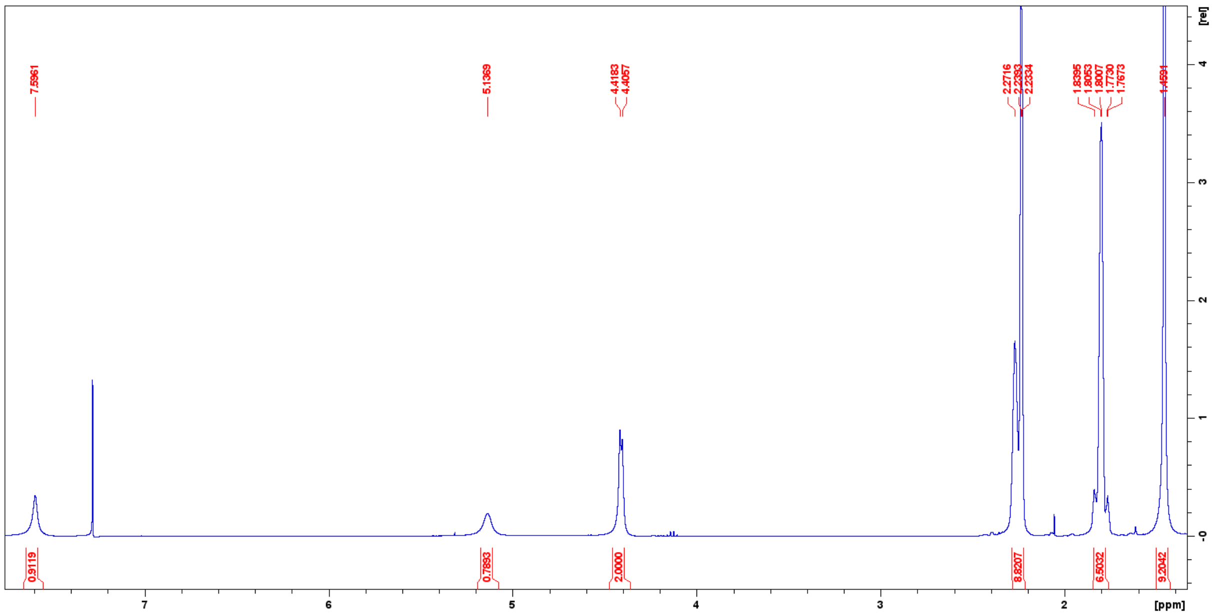

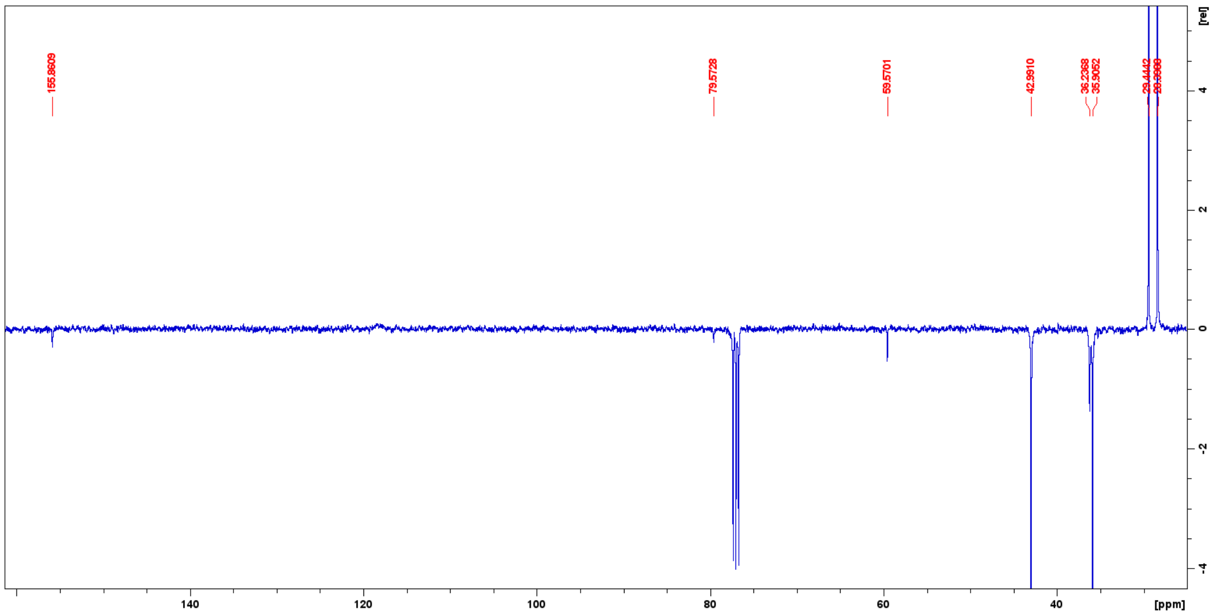

Figure S1. NMR spectra of compound **1a**

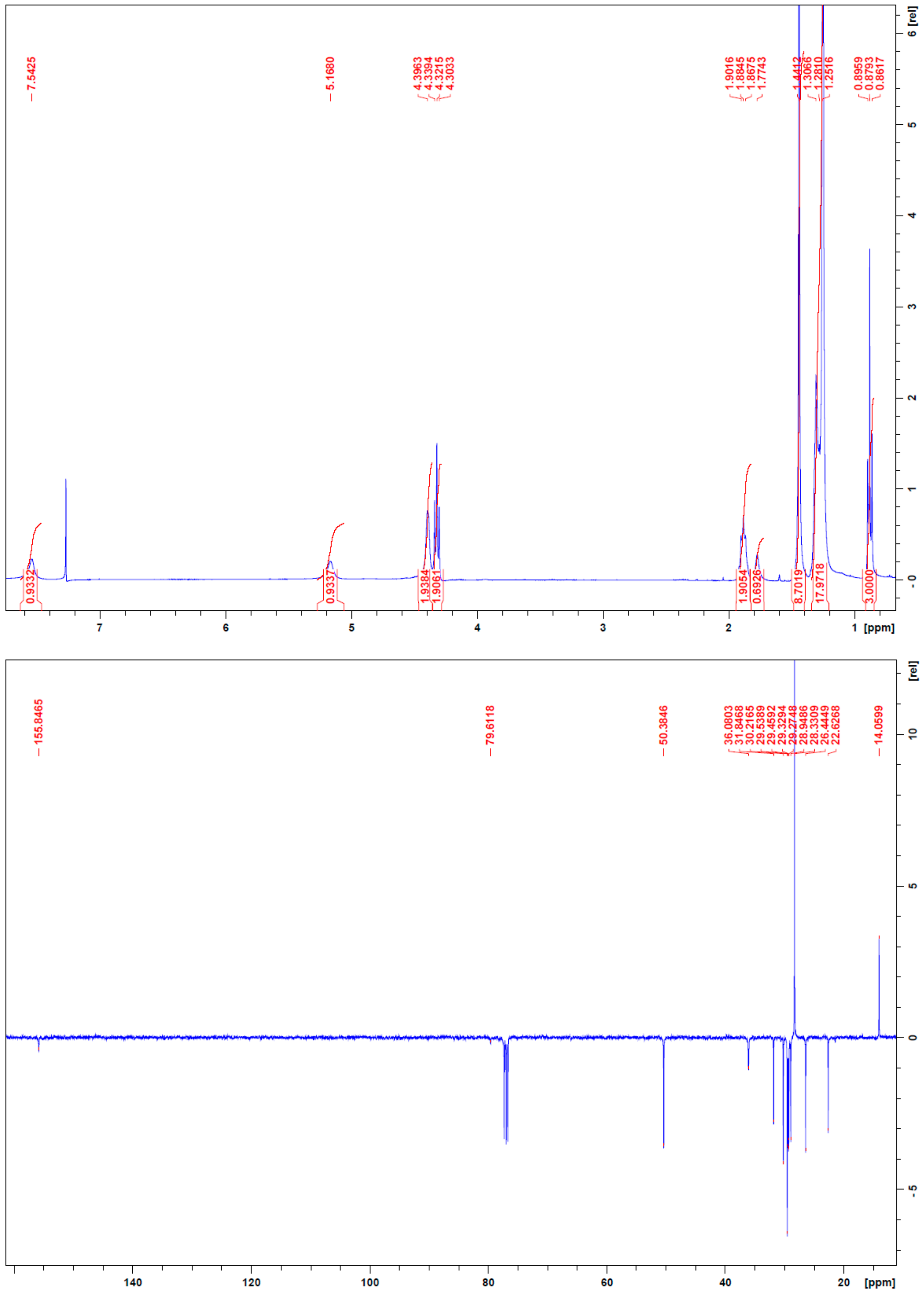

Figure S2. NMR spectra of compound **1b**

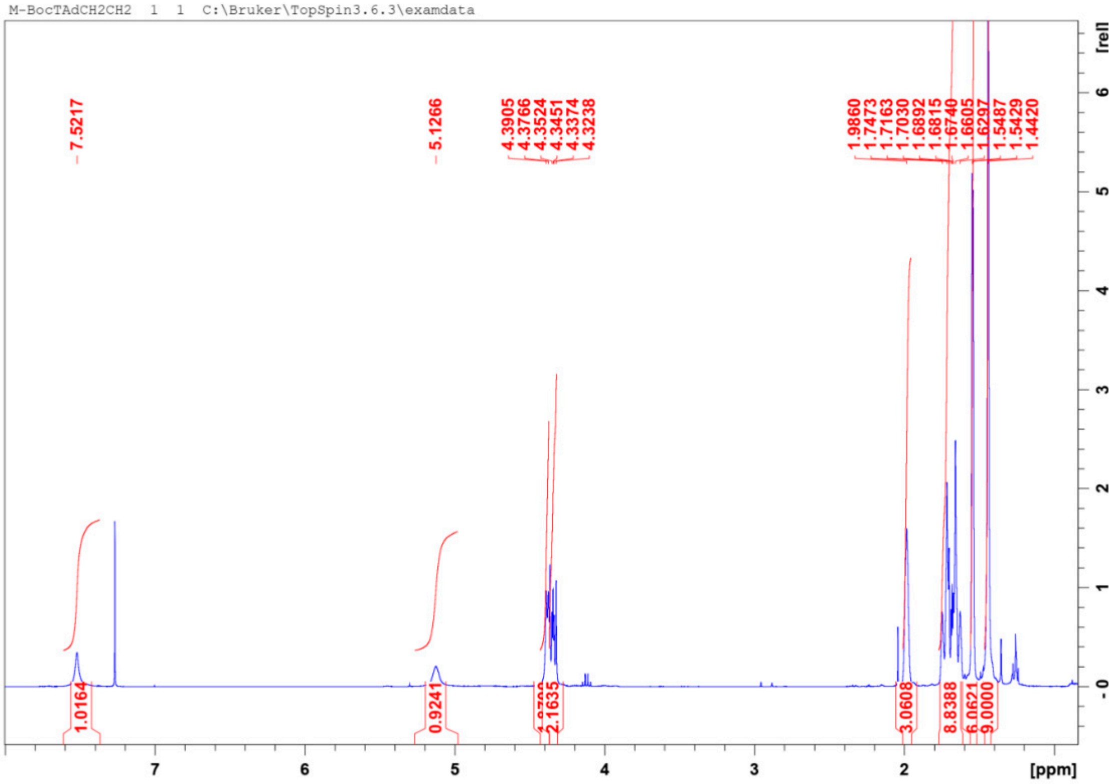

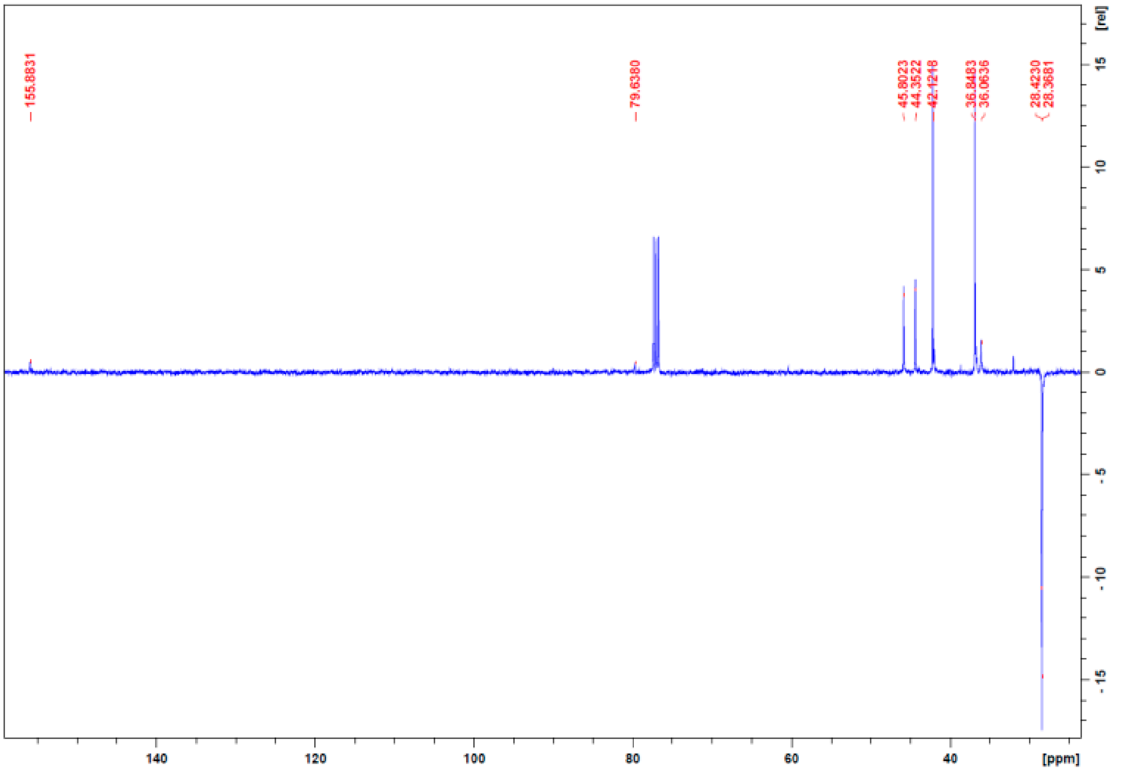

Figure S3. NMR spectra of compound **1c**

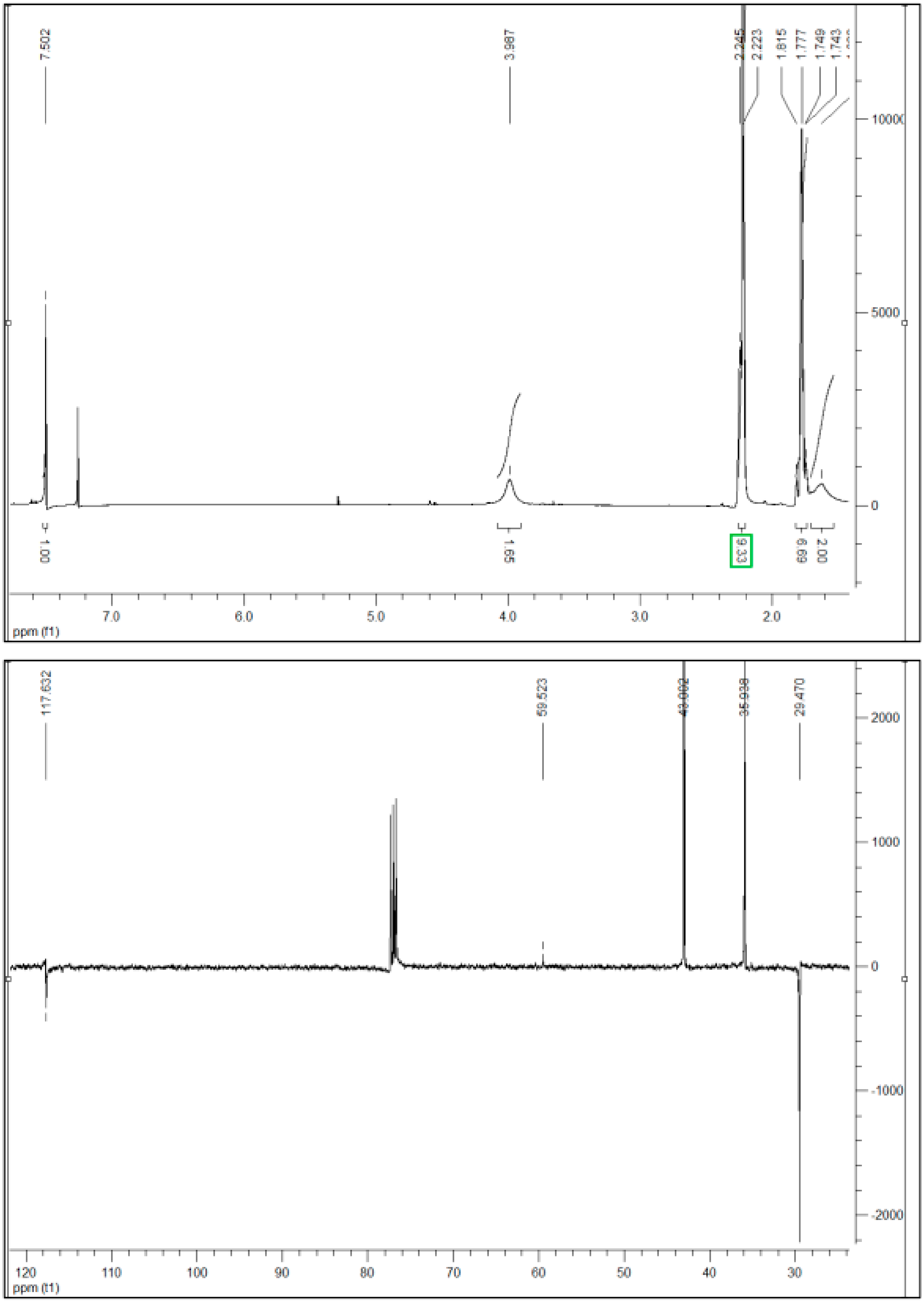

Figure S4. NMR spectra of compound **2a**

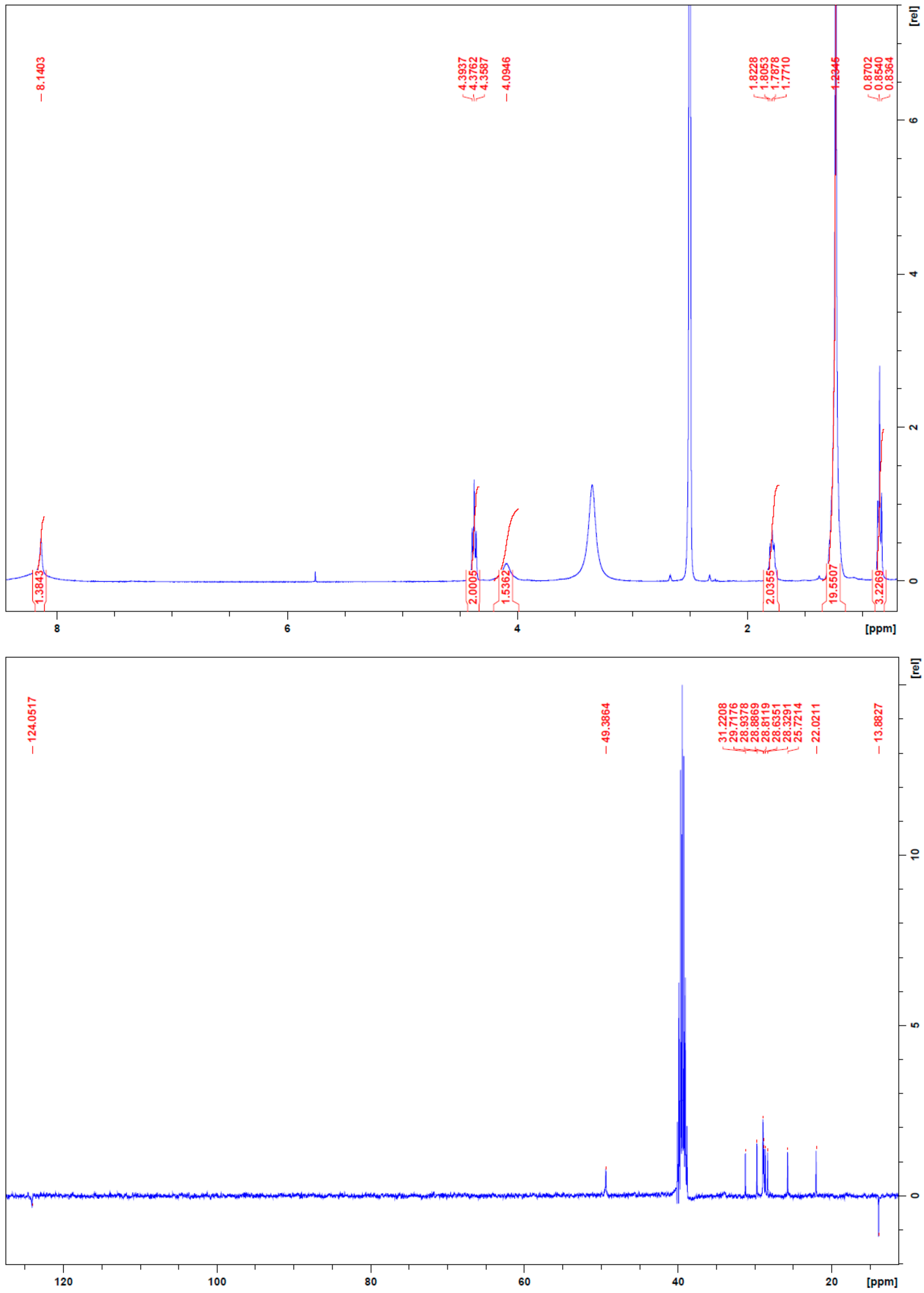

Figure S5. NMR spectra of compound **2b**

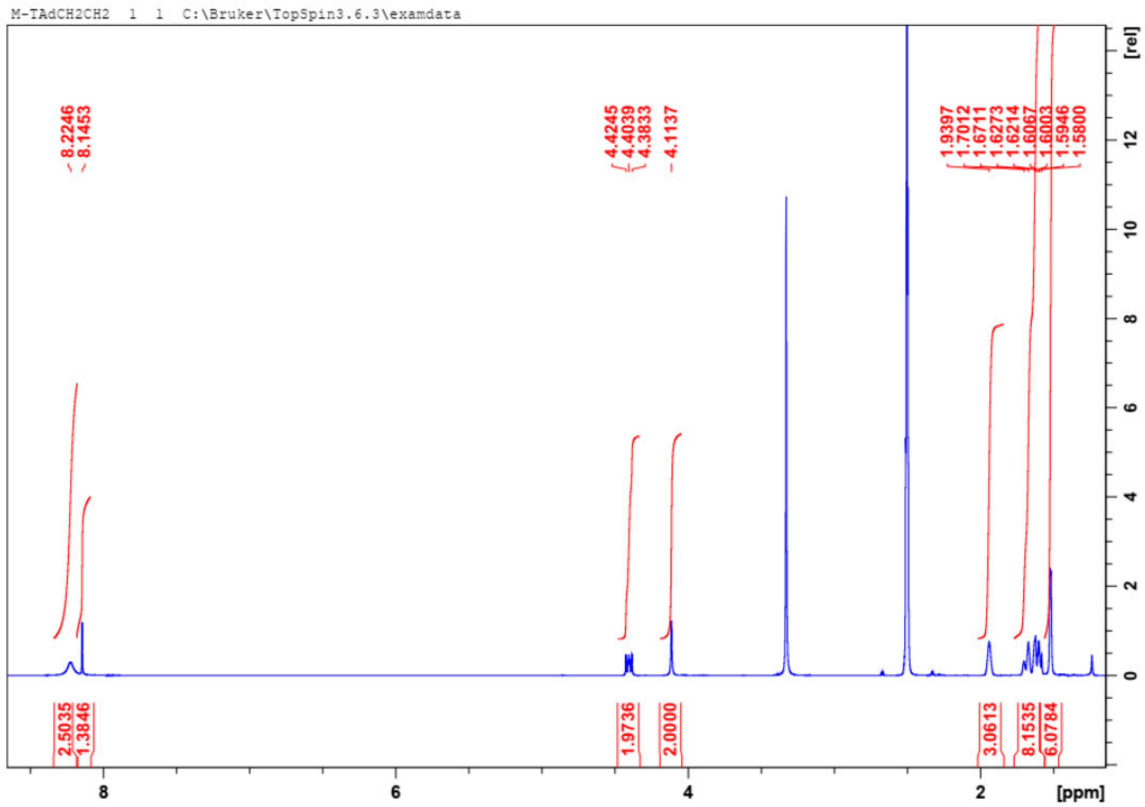

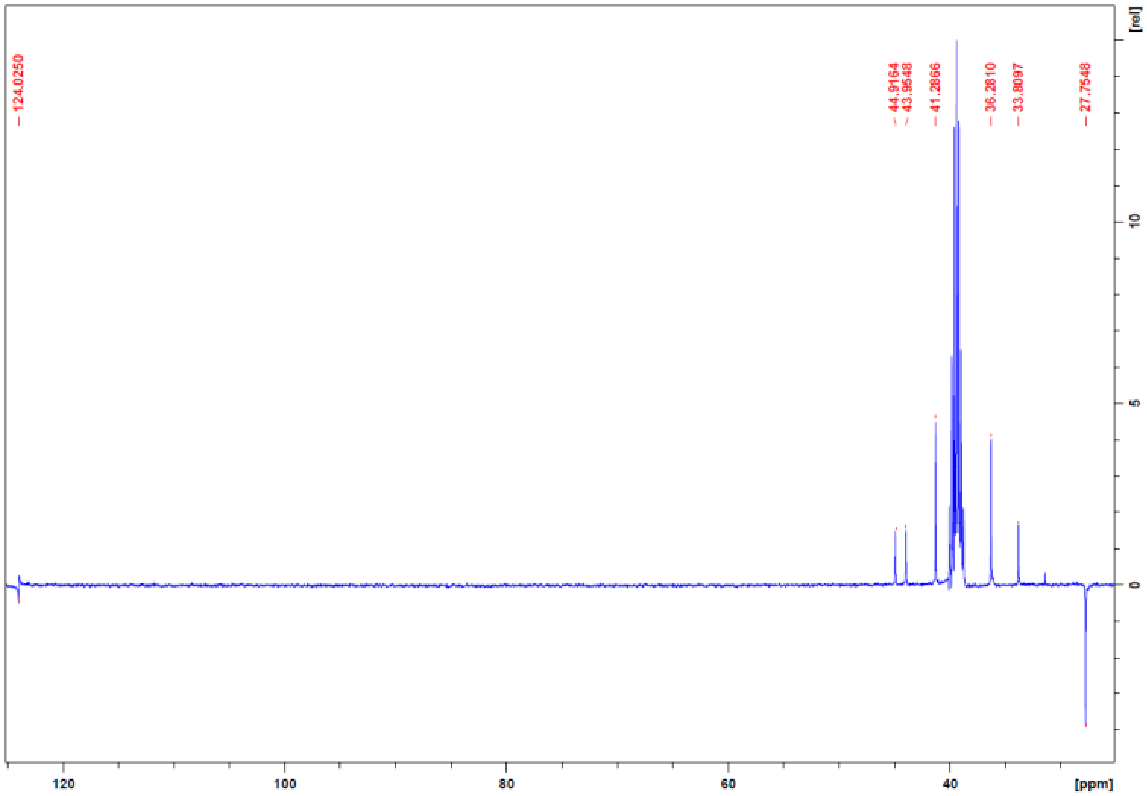

Figure S6. NMR spectra of compound **2c**

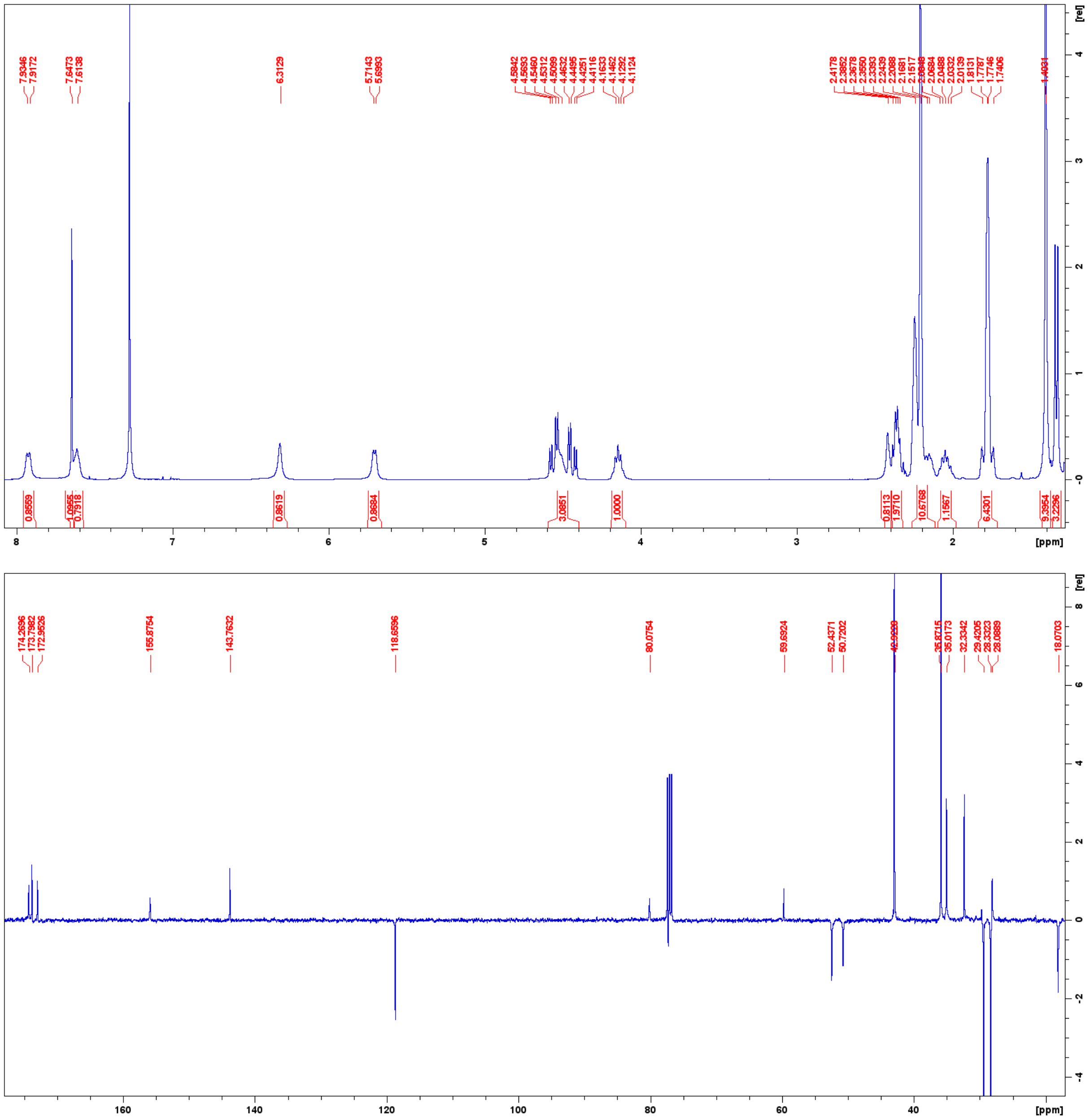

Figure S7. NMR spectra of compound **3a**

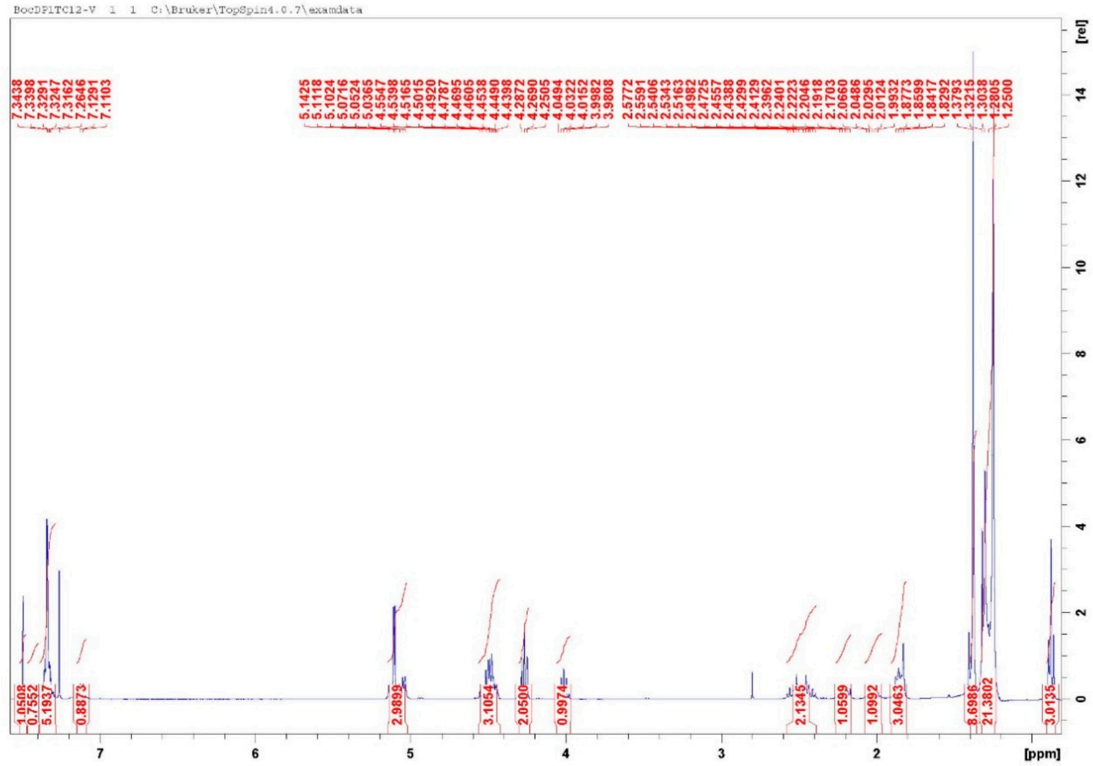

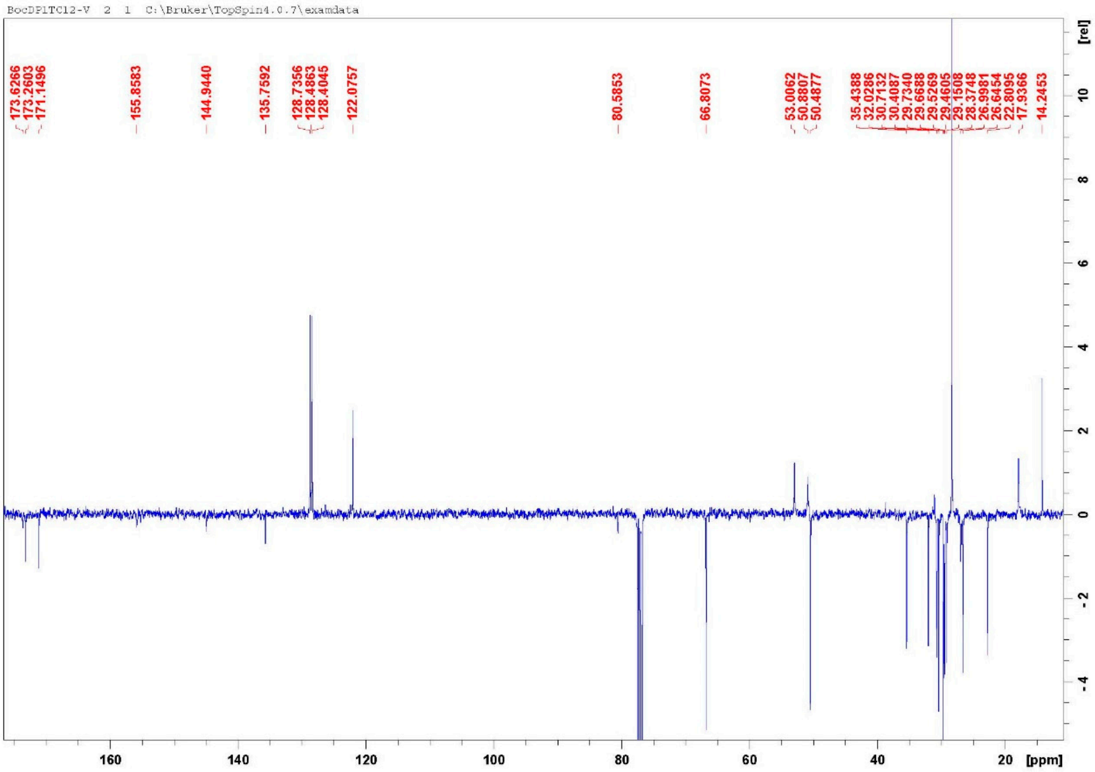

Figure S8. NMR spectra of compound **3b**

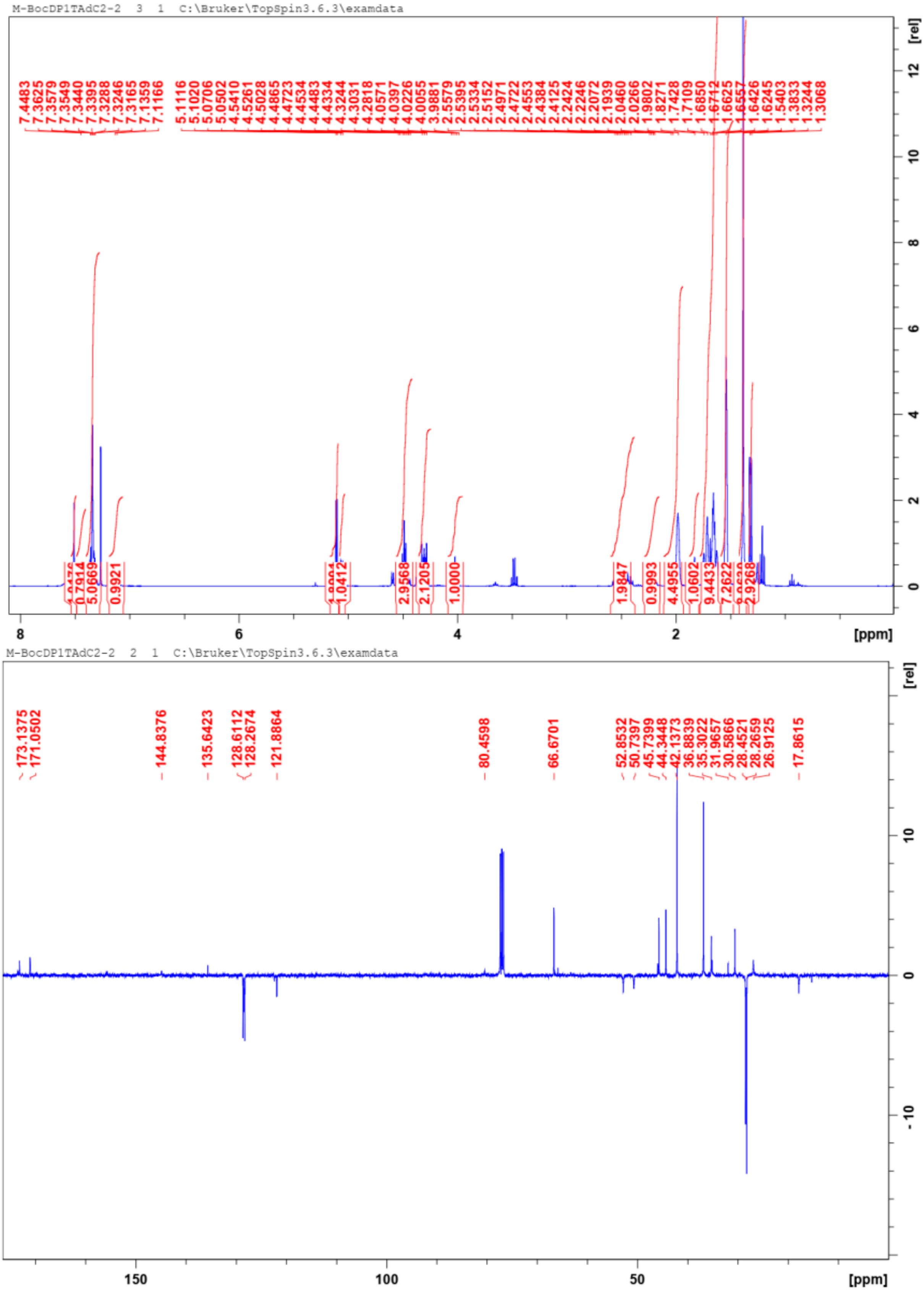

Figure S9. NMR spectra of compound **3c**

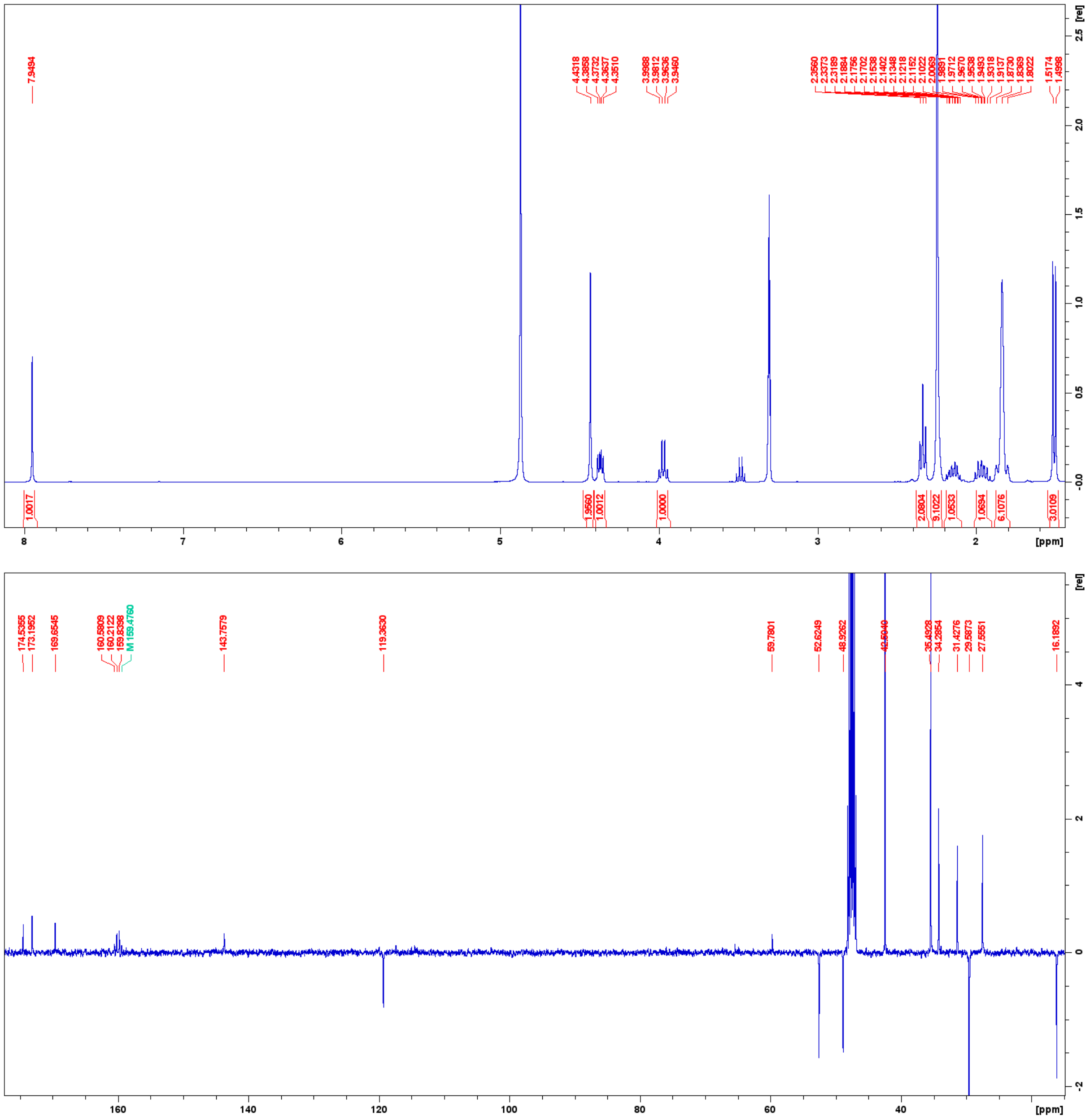

Figure S10. NMR spectra of compound **4a**

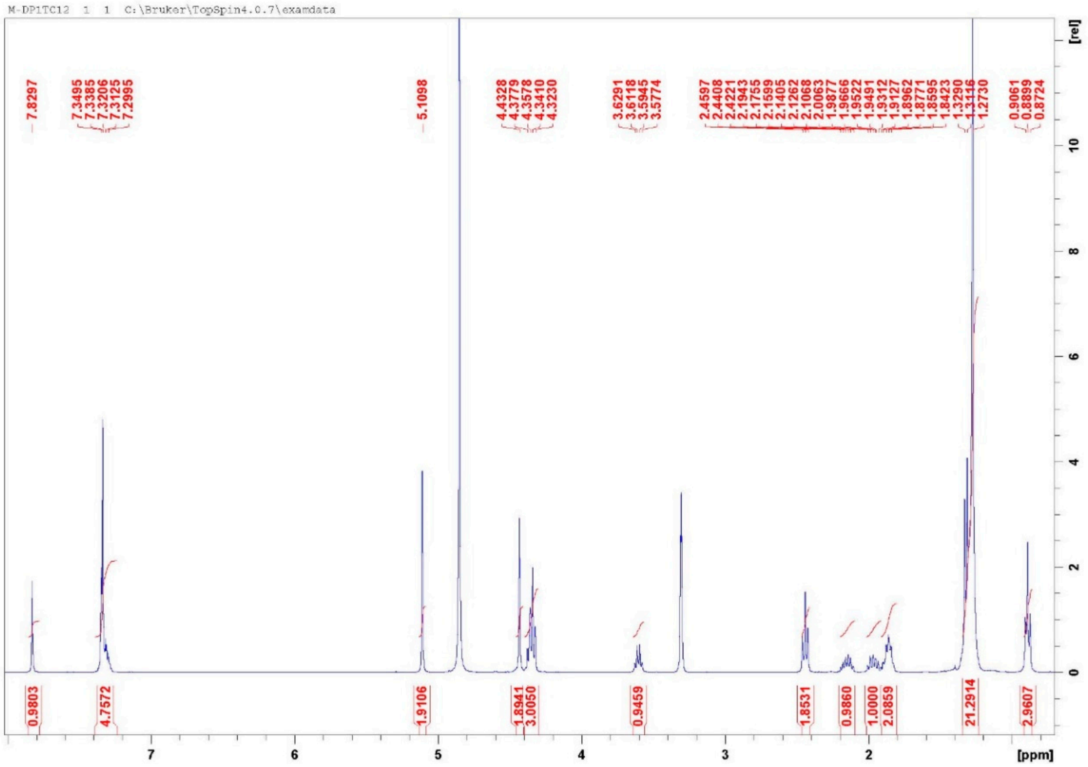

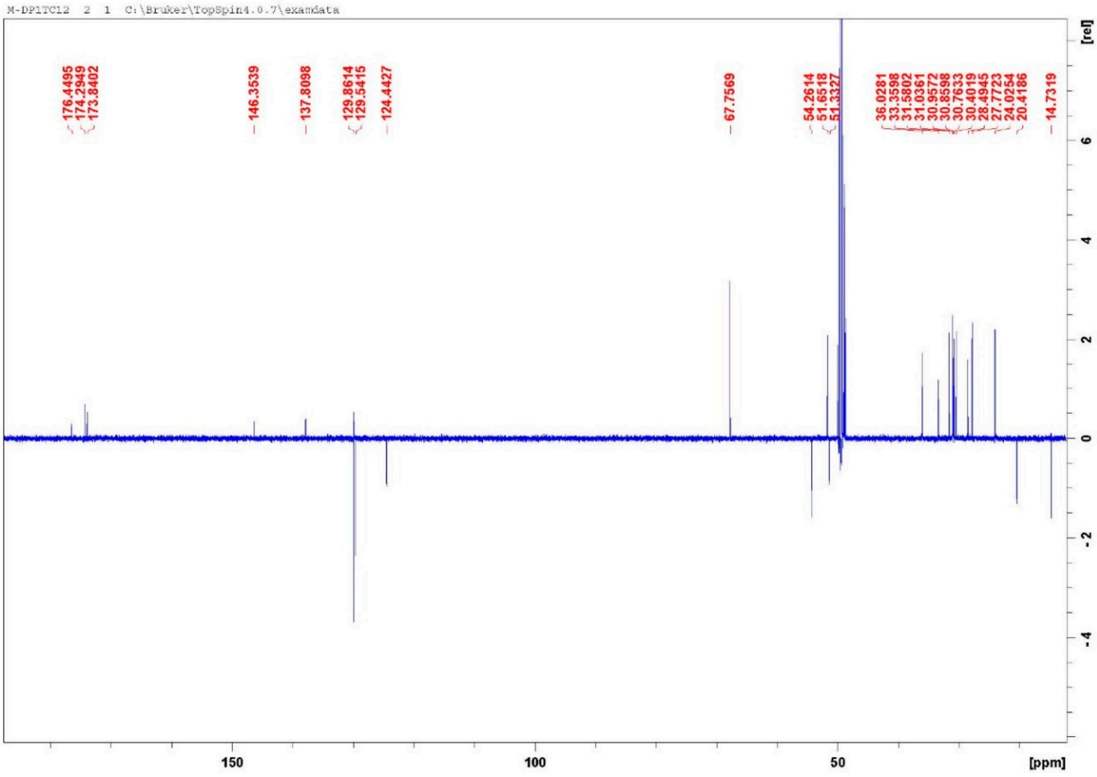

Figure S11. NMR spectra of compound **4b**

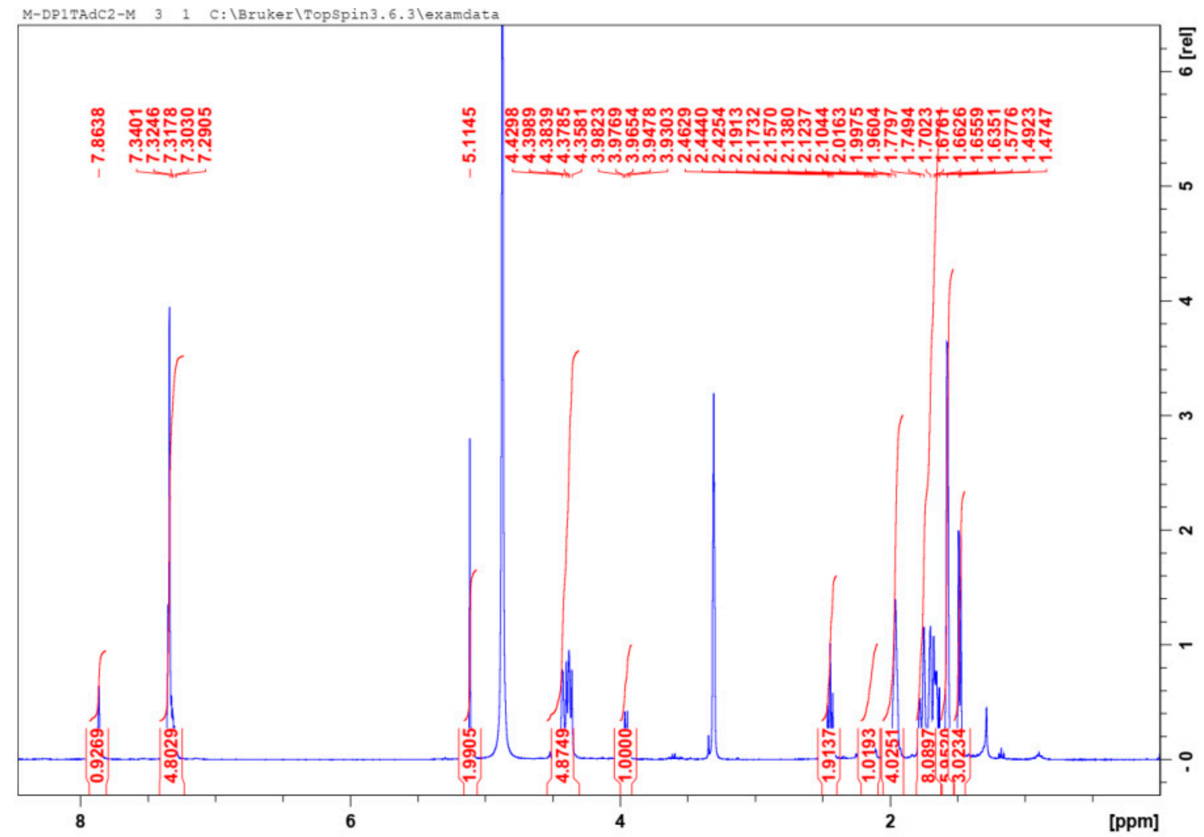

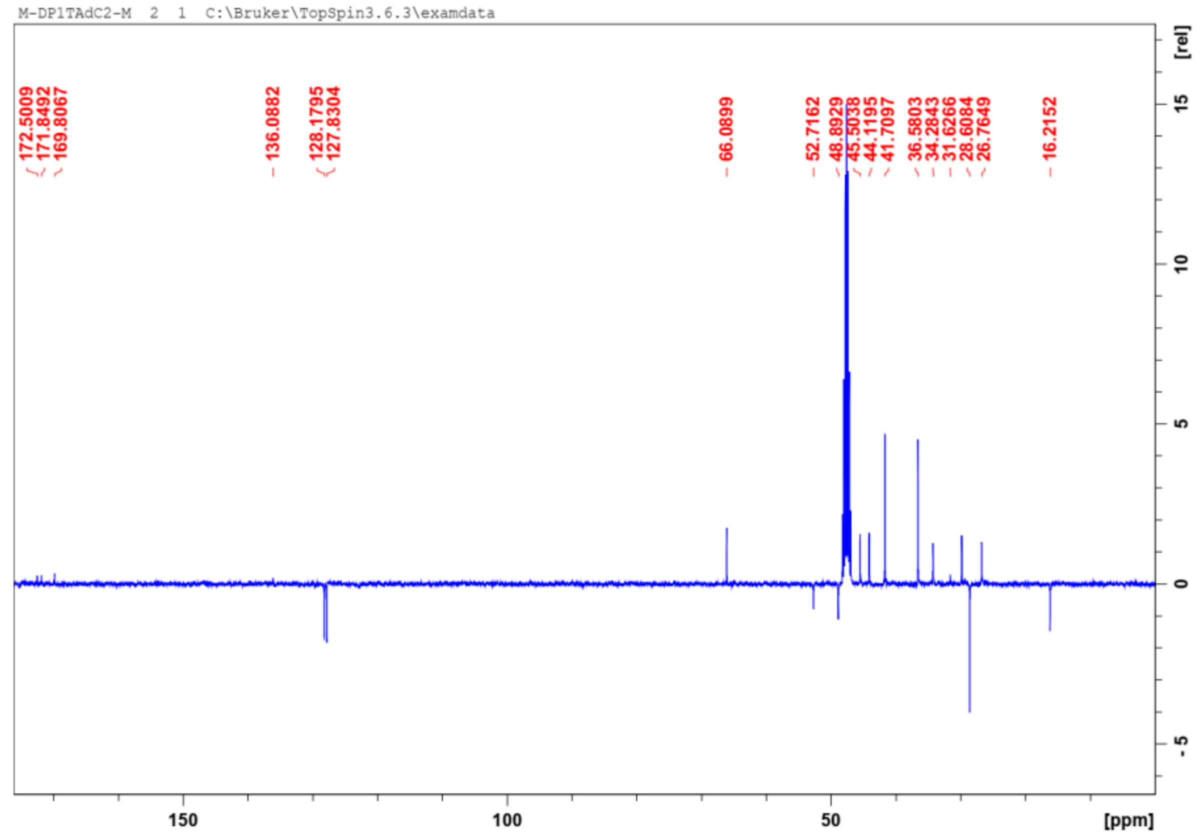

Figure S12. NMR spectra of compound **4c**

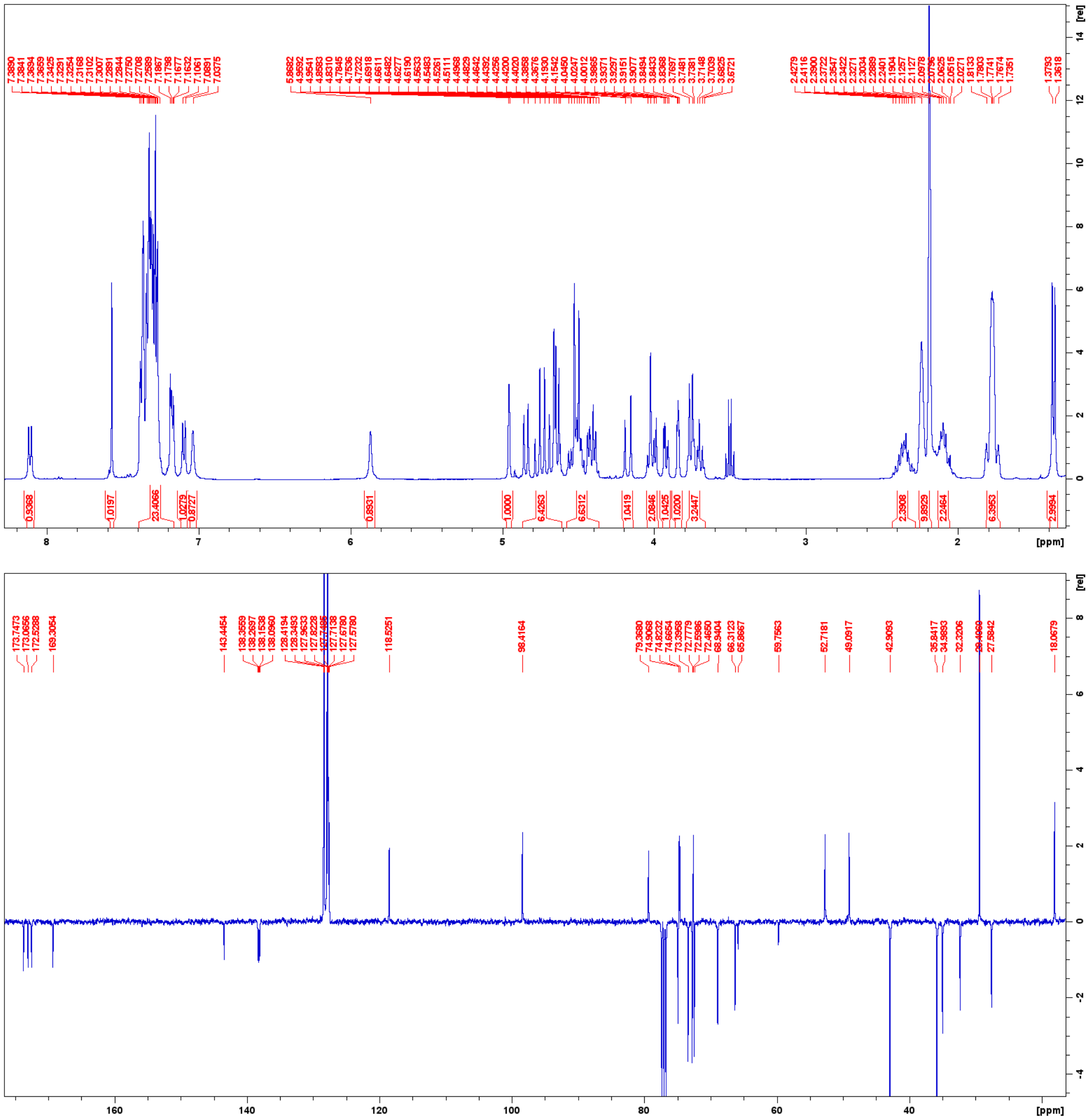

Figure S13. NMR spectra of compound **5a**

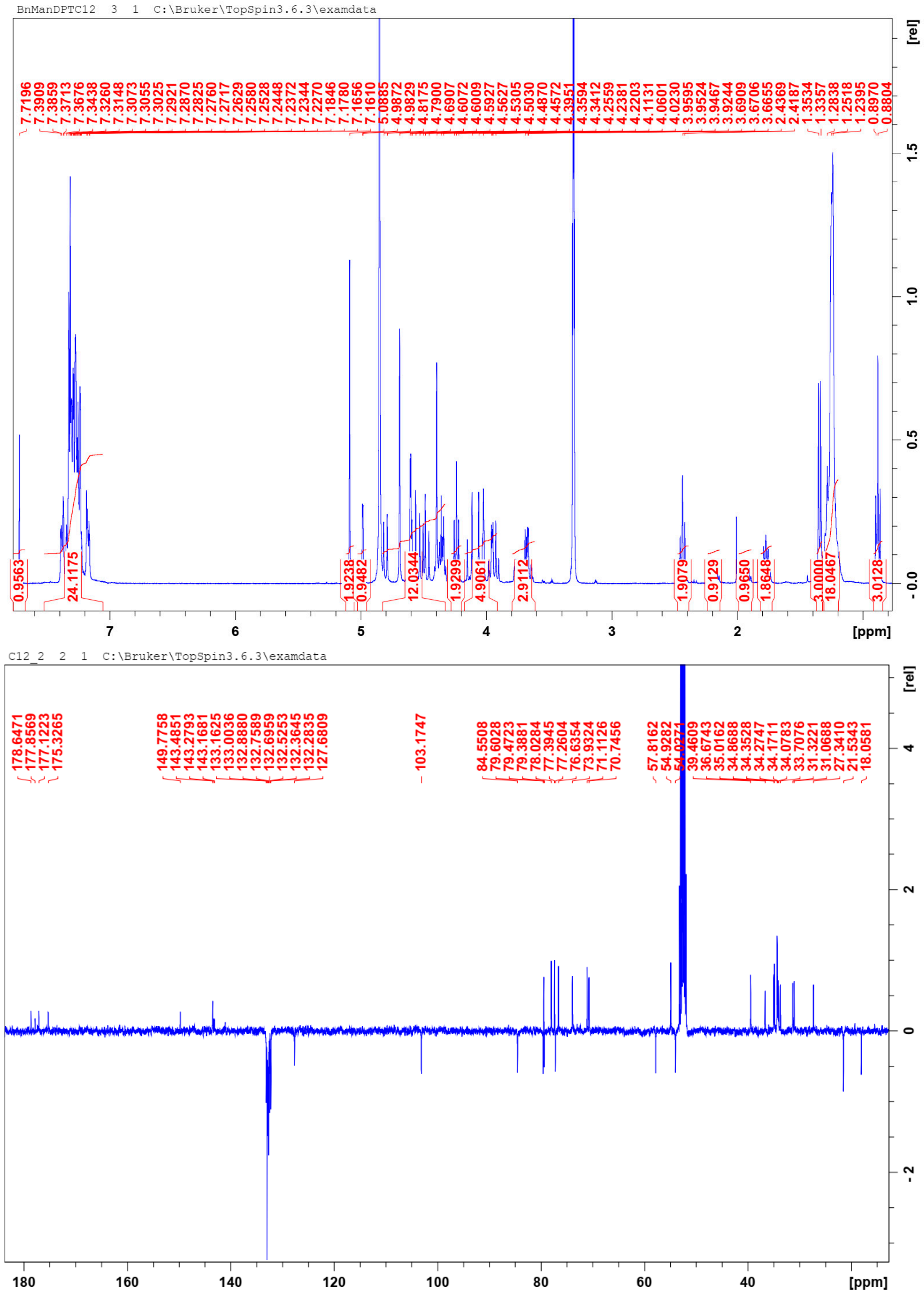

Figure S14. NMR spectra of compound **5b**

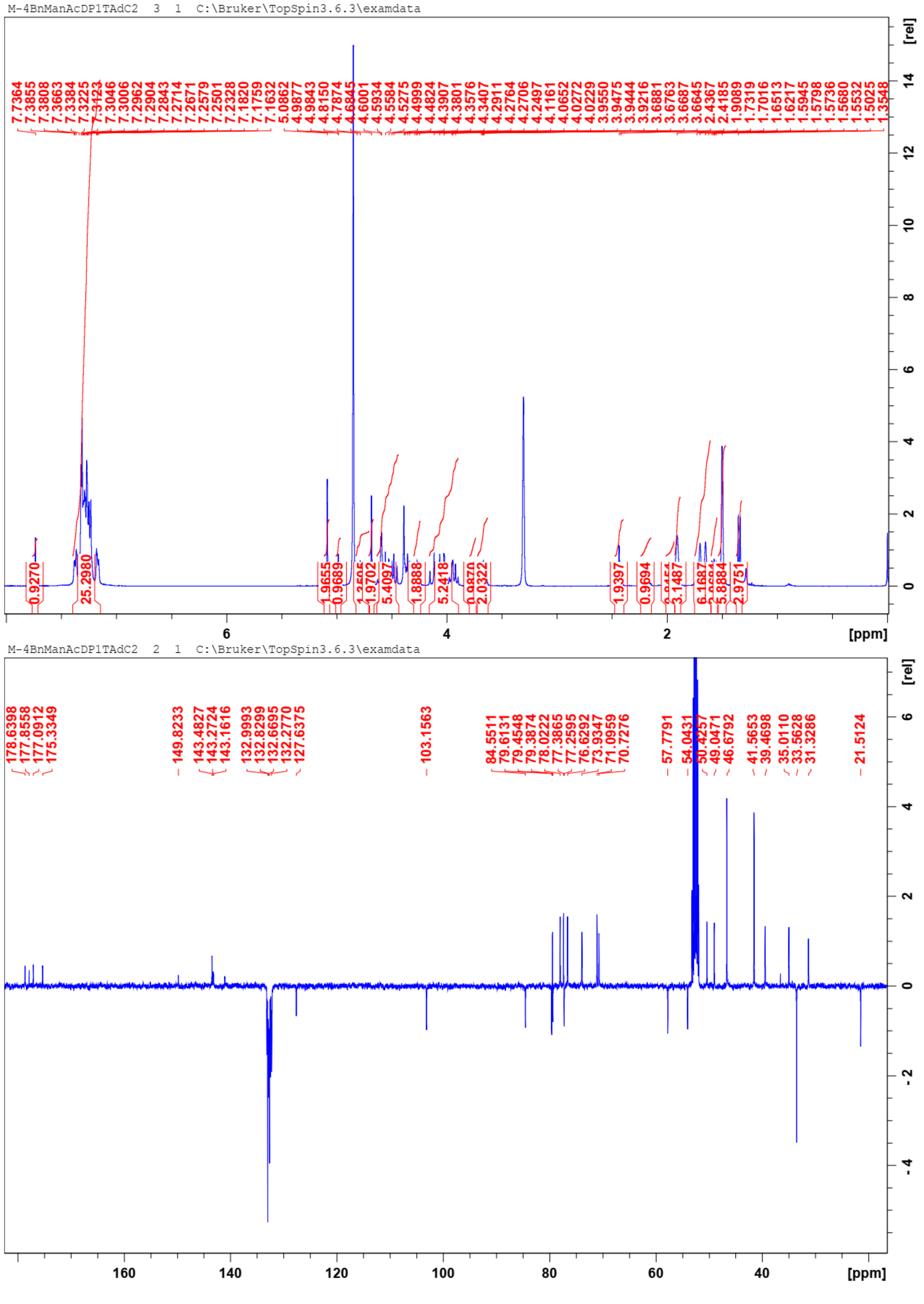

Figure S15. NMR spectra of compound **5c**

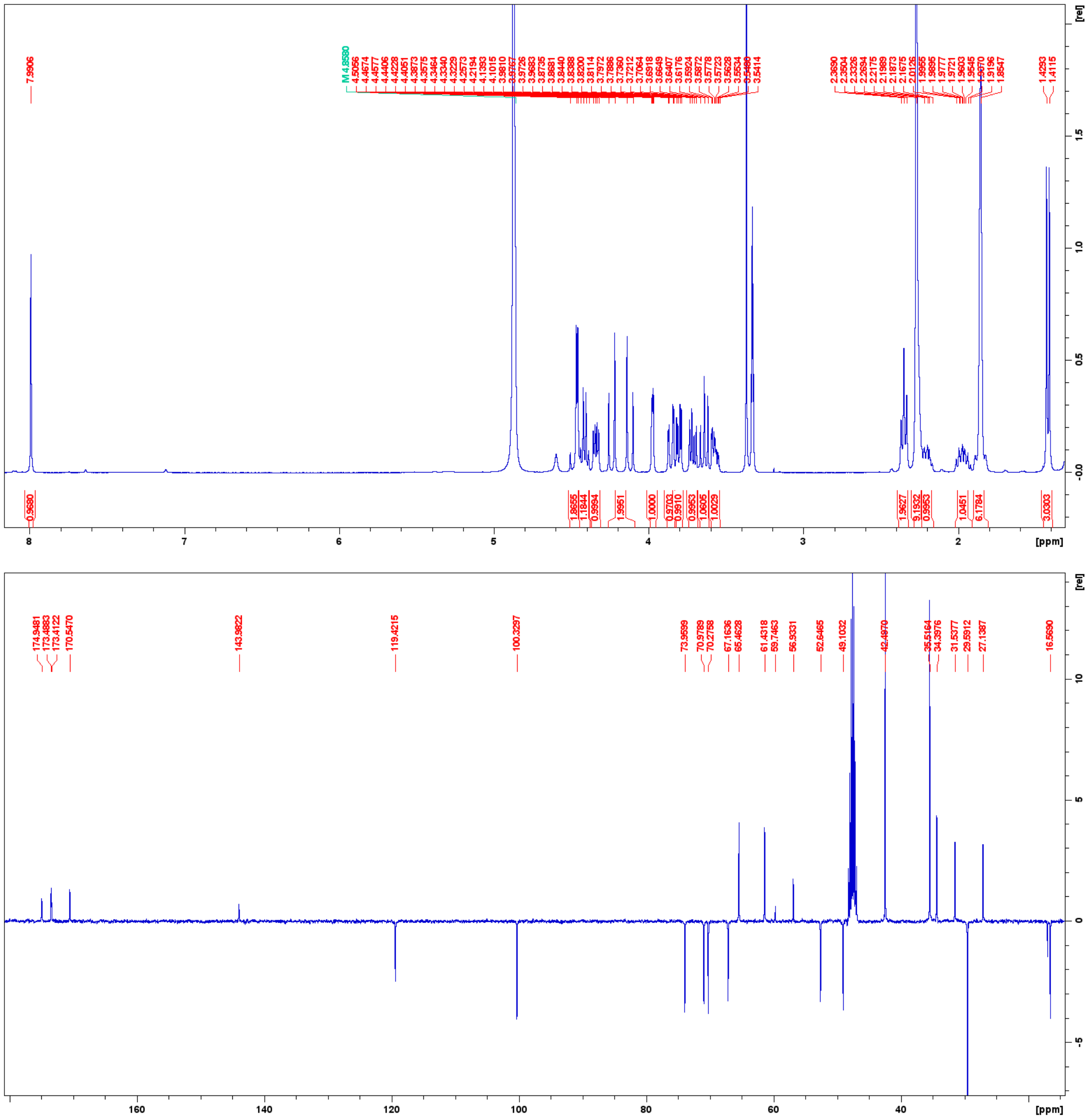

Figure S16. NMR spectra of compound **6a**

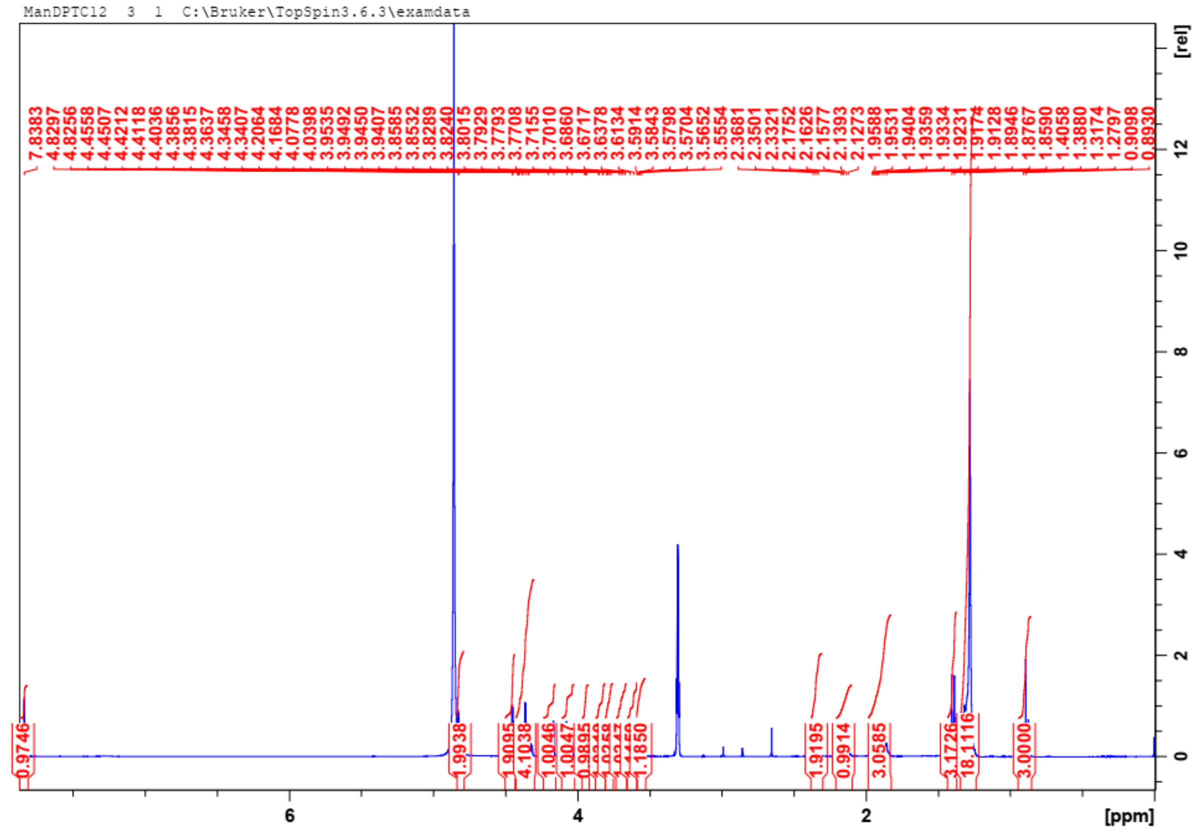

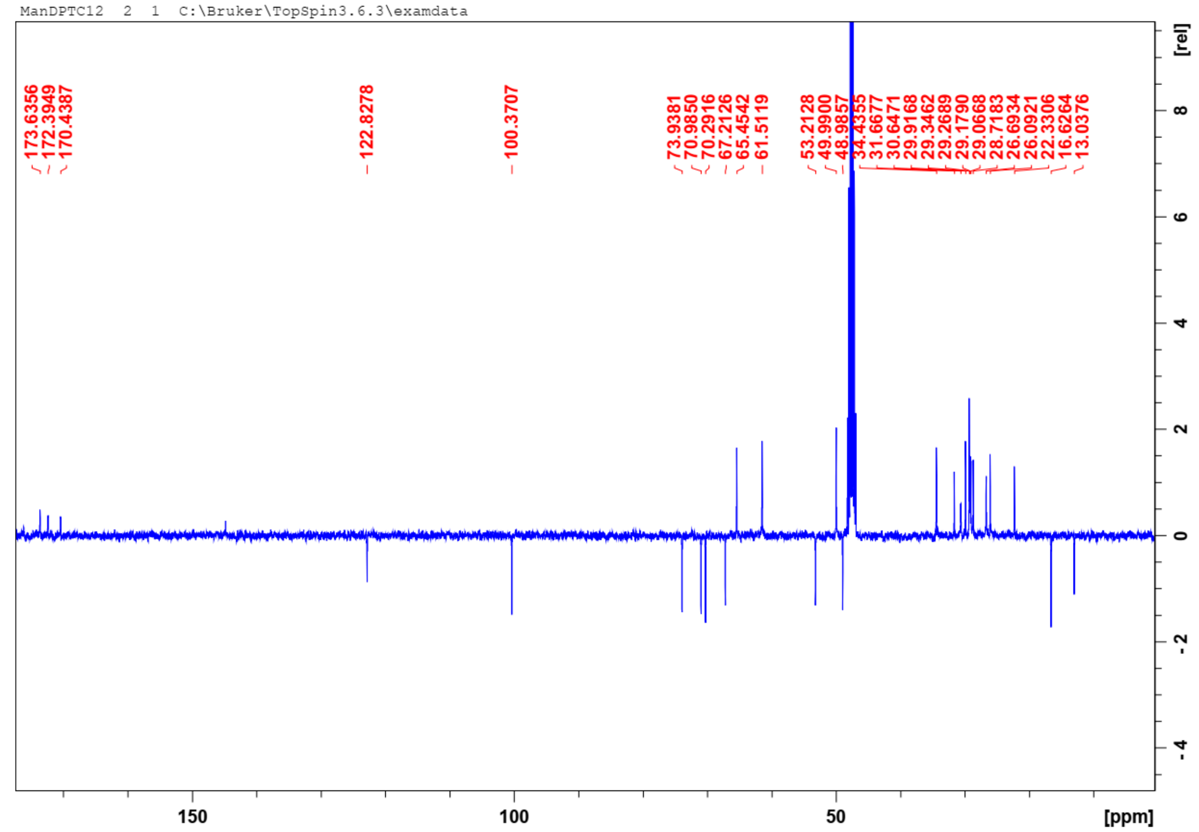

Figure S17. NMR spectra of compound **6b**

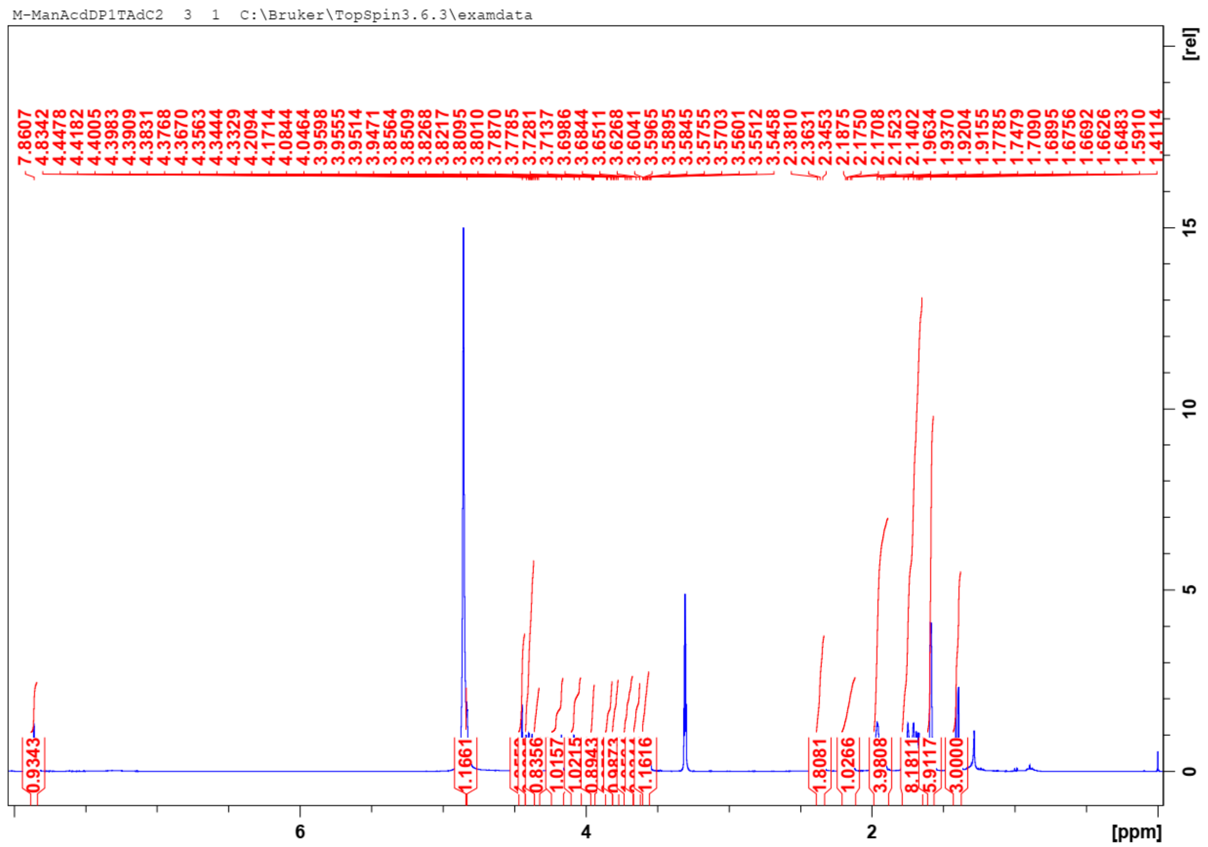

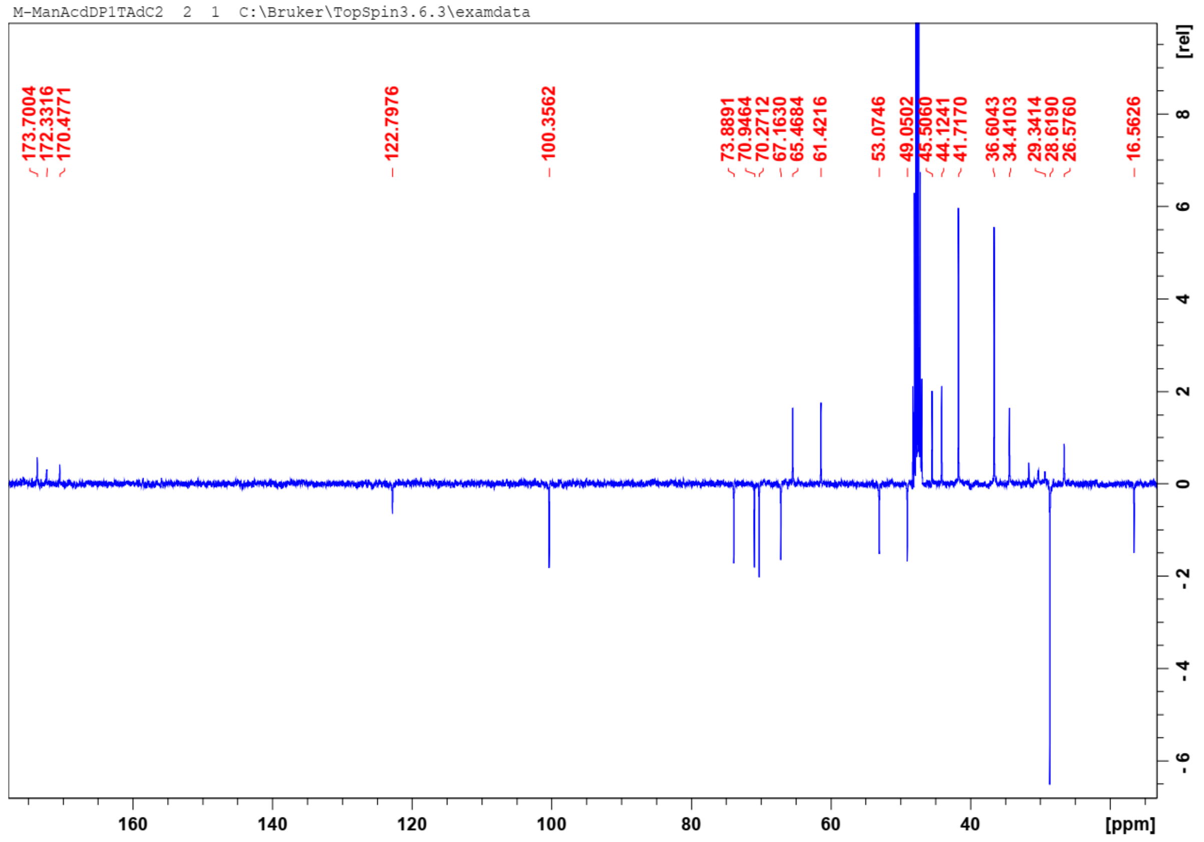

Figure S18. NMR spectra of compound **6c**

Table S1. Binding energy scores of synthesized DMPs.

Compound	Dock Score (kcal/mol)
**ManDMPTAd**	-8.2
**6b**	-6.7
**6c**	-8.0
